# Three-dimensional-printed ROS-scavenging and immunomodulatory hydrogel accelerates diabetic wound healing through synergistic microenvironment regulation

**DOI:** 10.1093/rb/rbag066

**Published:** 2026-03-28

**Authors:** Xin Cao, Yang Wang, Yuanhang Xu, Nai Liang, Jiabao Xu, Xinyun Li, Cong Ye, Caichou Zhao

**Affiliations:** Department of Dermatology, Affiliated Hospital of Nantong University, Medical School of Nantong University, Nantong 226001, China; Affiliated Hospital of Hebei University, Baoding 071000, China; The Third People’s Hospital of Qidong, Qidong 226200, China; Dongyang People’s Hospital, Dongyang 322100, China; Maternal and Child Health Hospital of Changxing County, Changxing 313100, China; Affiliated Hospital of Nantong University, Medical School of Nantong University, Nantong 226001, China; Affiliated Hospital of Nantong University, Medical School of Nantong University, Nantong 226001, China; Department of Dermatology, Affiliated Hospital of Nantong University, Medical School of Nantong University, Nantong 226001, China; Department of Dermatology, Affiliated Hospital of Nantong University, Medical School of Nantong University, Nantong 226001, China

**Keywords:** diabetic wound healing, nanoparticle, immunomodulation, angiogenesis, 3D-printing, hydrogel

## Abstract

The management of diabetic wounds remains clinically challenging, primarily owing to three interrelated pathological mechanisms: excessive accumulation of reactive oxygen species (ROS), dysregulated angiogenesis and sustained, non-resolving inflammation. To address these issues, we developed a multifunctional 3D-printed nanoparticle enhanced bionic skin (3D-NEBS) by integrating polydopamine-gallium-arginine nanoparticles (PDA-Ga-Arg) into a self-healing hydrogel matrix composed of oxidized *Tremella fuciformis* polysaccharide, carboxymethyl chitosan and fish gelatin. The resulting dressing exhibited mechanical adaptability, pH-responsive release, robust ROS-scavenging capacity and targeted immunomodulatory activity. *In vitro*, 3D-NEBS promoted M2 macrophage polarization, reduced inflammation, enhanced nitric oxide (NO) production and improved endothelial cell migration and tube formation. Broad-spectrum efficacy was confirmed, with significant growth inhibition observed against *S. aureus* and *E. coli*. In a diabetic rat model, 3D-NEBS achieved 92.79% ± 0.39% wound closure within 14 days, with enhanced collagen deposition and neovascularization. Transcriptomic analysis revealed upregulation of pathways related to collagen synthesis, antioxidant response and immune regulation. This study presents a synergistic hydrogel-nanoparticle platform with strong potential for diabetic wound repair.

## Graphical Abstract

**Graphical Abstract:**
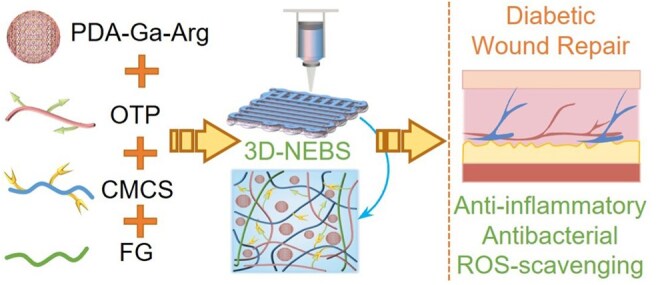

## Introduction

Diabetes represents a global public health crisis, with prevalence surging nearly 300% worldwide over the past three decades [1–3]. Of grave clinical concern, approximately 25% of individuals with diabetes develop chronic, non-healing cutaneous wounds [4–7]. These wounds are trapped in a vicious cycle characterized by persistent hyperglycemia, elevated oxidative stress and heightened bacterial susceptibility [8], rendering conventional therapies largely ineffective and leading to limb amputation in 20% of cases [9]. Current standard care suffers from two fundamental limitations: the inability to modulate the local pathophysiological microenvironment and the lack of personalized treatment strategies. Therefore, the design and fabrication of intelligent wound dressings with dynamic responsiveness and multifunctional therapeutic capabilities has become an urgent medical priority [10–12].

The physiological wound healing cascade comprises four dynamically coordinated, sequential phases of hemostasis, inflammation, cell proliferation and remodeling [13, 14]. Diabetic foot ulcers (DFUs), however, typically stagnate in the inflammatory phase due to multifactorial molecular pathologies: (i) Hyperglycemia-driven accumulation of advanced glycation end products perpetuates inflammation via RAGE receptor activation [15, 16]; (ii) Mitochondrial dysfunction induces excessive ROS generation and oxidative damage [17, 18]; (iii) Suppressed VEGF expression severely compromises angiogenesis [19]; and (iv) Immune dysregulation featuring M1 macrophage polarization impedes inflammation resolution and tissue regeneration [20, 21]. These interconnected pathologies create a self-perpetuating vicious cycle [22, 23].

Functional hydrogel dressings have shown promise in addressing these challenges. An ideal dressing should: maintain optimal moisture while permitting gas exchange; exhibit mechanical properties matching dynamic wound conditions; enable controlled therapeutic delivery; and possess microenvironment-responsive regulation capabilities [24, 25]. Natural polysaccharide-based systems are particularly advantageous due to their superior biocompatibility and biodegradability.

To address these challenges, we hypothesize that advanced structural engineering of a natural polysaccharide-based dynamic hydrogel could provide a comprehensive solution. The basidiomycete fungus *Tremella fuciformis* polysaccharide (TP) was selected as the base material due to its renowned moisturizing capacity [26, 27], potent antioxidant activity and demonstrated anti-inflammatory effect [28–30]. Its molecular structure—comprising Fucp:Xylp:Manp:GlcAp (0.9:1.0:3.2:1.2)—confers carboxyl-rich anionic characteristics and direct free radical scavenging capacity attributable to abundant hydroxyl groups [31]. However, most existing hydrogel dressings face two technological bottlenecks: functional simplicity inadequate for complex therapeutic needs, and fabrication complexity hindering clinical translation.

Recent advances in nanomaterials have opened new avenues for diabetic wound therapy [31, 32]. Compared to natural enzymes, functional nanoparticles offer key advantages such as exceptional stability, low production cost and scalable manufacturability. Our innovatively designed polydopamine-gallium-arginine (PDA-Ga-Arg) nanoparticles serve as multifunctional active components through dual mechanisms: (i) Scavenging reactive oxygen species through surface-mediated redox reactions; (ii) Enhancing NO release via arginine metabolism to improve local circulation. For efficient delivery, we engineered a novel 3D-printable self-healing hydrogel system featuring: (i) Dynamic oxidized *Tremella* polysaccharide (OTP)-CMCS Schiff base networks enabling initiator-free crosslinking, shear-thinning behavior and rapid self-healing; (ii) Fish gelatin (FG) providing RGD cell-adhesive motifs [33–35].

The key innovation of this study resides in the precise integration of macroscopic manufacturing with microscopic functionality through advanced structural engineering. First, we constructed a hydrogel network with autonomous self-healing capability using Schiff base dynamic covalent bonds, ensuring the dressing’s durability and integrity when applied to mobile areas like joints. Second, this dynamic network exhibits pH-responsive characteristics, enabling accelerated degradation in the acidic microenvironment typical of diabetic wounds, thereby facilitating faster release of inherent active components (such as the antioxidant fragments of OTP) for “on-demand” therapy. Most crucially, we incorporated 3D printing technology to fabricate this active hydrogel into customizable scaffolds. The regular, porous structure not only facilitates gas exchange and exudate management but also provides a biomimetic three-dimensional support for cell infiltration and tissue regeneration—advantages unattainable with traditional dressings or disordered hydrogels.

In this study, we developed a 3D-printed nanoparticle enhanced bionic skin (3D-NEBS). While individual components such as polydopamine, gallium or arginine have been explored previously, our work introduces several key advances: (i) A novel ternary nanoparticle design: The PDA-Ga-Arg nanoparticle uniquely integrates ROS scavenging, NO donation and antibacterial functions into a single entity, creating a self-amplifying therapeutic cycle that simultaneously addresses oxidative stress, impaired angiogenesis and infection. (ii) A structurally intelligent delivery platform: Beyond being a simple carrier, the OTP-based dynamic hydrogel exhibits autonomous self-healing and pH-responsive degradation, ensuring mechanical durability and “on-demand” therapeutic release in the acidic wound microenvironment. (iii) Personalized fabrication via functional 3D printing: Leveraging the hydrogel’s shear-thinning and rapid self-healing properties, we employ extrusion-based 3D printing to fabricate dressings with customizable architectures that conform perfectly to wound contours, providing a biomimetic 3D porous structure conducive to tissue integration—a significant advantage over conventional cast hydrogels. Through comprehensive *in vitro* and *in vivo* evaluations coupled with transcriptomic analysis, we demonstrate that 3D-NEBS acts not merely as a combination of parts, but as a synergistic system that coordinately reprograms the diabetic wound microenvironment, offering a distinct and effective strategy beyond existing single-function approaches.

## Materials and methods

### Materials

*Tremella fuciformis* polysaccharide (TFP), a naturally occurring acidic heteroglycan, was sourced from Macklin Biochemical Co., Ltd. (Shanghai, China). Structural analysis reveals that its main chain is built from β-D-xylopyranose, α-D-mannopyranose and α-D-glucuronic acid units, primarily connected via α-(1 → 3)-glycosidic linkages; branched motifs include β-D-galactopyranose, α-L-arabinofuranose and minor quantities of α-L-rhamnopyranose. Quantitative monosaccharide composition yields a molar ratio of Xylp:Manp:GlcAp:Galp = 0.9:1.0:3.2:1.2. The polymer exhibits a weight-average molecular weight of ∼1.0 × 10^6^ Da, an intrinsic viscosity of no less than 1.2 × 10^4^ mPa·s (measured in 0.1 M NaCl at 25°C) and a purity exceeding 90%, as confirmed by high-performance liquid chromatography (HPLC). Its CAS number is 9075-53-0. Carboxymethyl chitosan (CMCS), with a molecular weight of ∼3.0 × 10^5^ Da, intrinsic viscosity ≤ 100 mPa·s, purity ≥ 80% and degree of substitution ≥ 80%, was also acquired from Macklin Biochemical Co., Ltd. Fish gelatin (FG), extracted from skins of cold-water fish species, was obtained from Sigma-Aldrich Co., LLC (St. Louis, MO, USA). Additional reagents—gallium nitrate, L-arginine, Pluronic^®^ F127, dopamine hydrochloride and 1,3,5-trimethylbenzene—were procured from Aladdin Reagents (Shanghai, China). Enzymatic and oxidative stress assay kits (catalase activity, hydroxyl radical scavenging), DMEM, FBS, PBS, STZ (≥98%), LPS (O111: B4), sodium citrate, citric acid monohydrate, custom curing molds (1 cm inner diameter), HUVECs and RAW264.7 cells were obtained from Macklin, Gibco™, Yuanye, Suzhou Yongqinquan, and Oricell^®^, respectively.

### Synthesis and characterization of PDA-GA-LARG nanoparticles

Dopamine hydrochloride (90 mg) and gallium nitrate hydrate (10 mg) were dissolved in 3 mL of ultrapure water, and pH was adjusted to 8.0 with 0.1 M NaOH. After stirring at room temperature (25°C) for 24 h, a clear, stable dopamine–gallium coordination complex solution was obtained. In parallel, Pluronic^®^ F127 (36 mg) was fully dissolved in 6 mL anhydrous ethanol and 6 mL ultrapure water, aided by mild heating (40°C) and vortex agitation. Then, 0.6 mL of the chelate solution was introduced gradually into the F127 mixture under constant stirring (600 rpm) for 20 min to achieve homogeneous incorporation. Next, under controlled ultrasonication (40 kHz, 120 W; ice bath maintained at 10–15°C), 0.3 mL of TMB solution (10 mg/mL in ethanol; Aladdin Reagents, Shanghai, China) was infused over 2 min, followed by rapid mechanical shaking for 4 min to generate a uniform milky emulsion. Subsequently, 1 mL of L-arginine solution (9 mg/mL in ultrapure water) was added, and the mixture was incubated in the dark at 25°C for 6 h to enable oxidative polymerization and nanoparticle assembly. The colloidal product was then centrifuged at 4°C (12 000×*g*, 15 min). The pellet was rinsed once with anhydrous ethanol and twice with deionized water to eliminate residual reagents and surfactant. Purified nanoparticles were freeze-dried and stored desiccated at −20°C under a nitrogen atmosphere.

Material characterization was carried out using the following techniques: FTIR spectroscopy (Thermo Scientific Nicolet iS50, KBr pellet mode), UV–Vis absorption spectroscopy (Techcomp UV-1050, Shanghai, China), XPS analysis (ESCALAB 250Xi, USA) and TEM (Thermo Scientific Talos F200X, accelerating voltage: 200 kV).

### Cytocompatibility assessment of PDA-GA-LARG nanoparticles

HUVECs were plated in 96-well plates and permitted to adhere for 24 h. After exposure to graded doses of PDA–Ga–L-Arg nanoparticles for 1 day, cells were washed with PBS and then treated with 10% CCK-8 reagent prepared in complete DMEM for 2 h at 37°C. Absorbance was measured at 450 nm (with 630 nm as the reference wavelength) using a BioTek Synergy H1 microplate reader.

### Synthesis and characterization of 3D-BS and 3D-NEBS

Dissolve the *Tremella* polysaccharide at a concentration of 2 wt% in deionized water. After complete dissolution in a 60°C water bath, add 1 g of sodium periodate and stir in the dark for 6 h. Then, under dark conditions, dialyze in deionized water for 3 days to obtain oxidized *Tremella* polysaccharide (OTP). Following its synthesis, OTP was analyzed by ^1^H NMR, which confirmed the existence of aldehyde groups. Prepare a 5% (w/v) solution of carboxymethyl chitosan (degree of substitution ≥90%), a solution of OTP at a specified mass fraction and a 5% (w/v) solution of FG in PBS buffer. Mix these solutions in a 1:2:1 ratio to obtain the CMCS/OTP/FG (COF) solution. Subsequently, PDA–Ga–L-Arg nanoparticles were incorporated into the COF solution at 5.0 mg/mL and homogenized via mild stirring for 30 min to afford the COF@PDA–Ga–L-Arg printable precursor. In the final 3D-printed NEBS hydrogel, the nanoparticle loading was 5.0 mg/mL, corresponding to approximately 0.5 wt% relative to the total hydrogel mass. This loading amount was selected based on a series of pre-experiments that evaluated the release kinetics, antioxidant efficiency and biocompatibility of the composite hydrogel. The STL model file was imported into a 3D bioprinter with integrated path-planning software. COF solution and COF@PDA–Ga–L-Arg solution were separately loaded into 5 mL syringes and extruded through a 27G nozzle to fabricate circular patches (10 × 1 mm). The resulting patches are designated as 3D-printed bionic skin (3D-BS) and 3D-printed nanoparticle enhanced bionic skin (3D-NEBS), respectively. The 3D-BS underwent pre-crosslinking prior to printing, and the resulting hydrogel patch is subsequently characterized using FTIR (Thermo Fisher, iS50) following lyophilization in a freeze dryer.

### Rheological analysis of 3D-NEBS

Rheological properties were evaluated using a rheometer, and tested by using an 8 mm stainless steel parallel-plate geometry. All tests were conducted in triplicate, and the storage modulus (*G*′) and loss modulus (*G*″) were quantified. In these investigations, *G*′and *G*″ were comprehensively characterized under controlled conditions: (i) frequency sweeps were conducted at 1% strain and 1 Hz to assess the viscoelastic response of hydrogel disks; (ii) strain sweeps were carried out at 1 Hz to evaluate the nonlinear mechanical behavior and yield point; and (iii) steady-state shear viscosity was measured over a shear rate range of 0.01–1000 s^−1^ at 25°C.

### Swelling and degradation test of 3D-NEBS

Swelling kinetics of 3D-NEBS were assessed using cylindrical samples (*d* = 5 mm, *h* = 4 mm). Dry weight (*W_d_*) was determined following ambient-air drying to constant mass. Wet weight (*W_w_*) was recorded at predetermined intervals. All measurements were repeated three times. The swelling ratio (%) was computed as described in Equation (1):


(1) $$\text{Swelling}\;\text{ratio}\;\left(\%\right)=\left(W_{w}-W_{d}\right)/W_{d}\times 100\%.$$

The degradation behavior of the 3D-NEBS hydrogel was evaluated by immersing 1 mL cylindrical specimens in 2 mL of PBS (pH 5.5, 6.5 or 7.4) and incubating them at 37°C under gentle horizontal shaking. Following equilibration of swelling, the initial weight (*W*_0_) was measured. At scheduled intervals, samples were carefully patted dry to remove excess surface moisture and promptly weighed to obtain the residual mass (*W*_1_). Weight retention (%) was then calculated using Equation (2):


(2) $$\text{Weight}\;\text{retention}\;(\%)=(W_{1}/W_{0})\times 100\%.$$

### Release kinetics of PDA-Ga-Arg nanoparticles from 3D-NEBS

To investigate the release kinetics of PDA-Ga-Arg nanoparticles from 3D-NEBS, 1 mL hydrogel specimens were immersed in 5 mL of PBS buffered to pH 5.5, 6.5 or 7.4 and incubated at 37°C under mild shaking. At each time point, the PBS was harvested and replenished with fresh buffer. All samples were in triplicate. The concentration of PDA-Ga-Arg nanoparticles was determined by UV–Vis spectrophotometry at 275 nm.

### Cytocompatibility of 3D-NEBS

HUVECs were seeded in 96-well plates and permitted to attach for 24 h before being treated with different hydrogel formulations for 1, 3 or 7 days. Following exposure, cells were rinsed thoroughly with PBS and incubated with 10% CCK-8 solution in culture medium at 37°C for 2 h; absorbance was then measured at 450 nm using a microplate reader. Additionally, a certain number of HUVEC cells were seeded into 48-well plates and cultured for 24 h. Sterile curing rings were placed, and sterilized hydrogels were placed on the curing rings for co-culture for 3 and 5 days. Following removal of the curing rings and hydrogel patches, cell viability was quantified using the Calcein-AM/PI dual-staining assay.

### DPPH scavenging experiment

According to previous reports, the DPPH radical scavenging assay was employed to evaluate the *in vitro* antioxidant activity of the hydrogels. Briefly, cylindrical samples specimens (10 mm in diameter and 5 mm thick) were immersed in freshly prepared DPPH solution (in ethanol). After 30 min of dark incubation at ambient temperature, the supernatant was harvested and analyzed at 517 nm by UV–Vis spectrophotometry to quantify DPPH radical scavenging activity.

### *In vitro* antibacterial properties of 3D-NEBS

The antimicrobial efficacy of the microparticles was assessed against *S. aureus* and *E. coli* via the agar plate colony enumeration assay. Briefly, standardized bacterial suspensions (2 × 10^6^ CFU mL^−1^) were co-incubated with equal volumes of sterile microparticle dispersions under aerobic conditions at 37°C with shaking at 120 rpm for 24 h. The control group consisted of suspensions without samples, with all solutions diluted 1000-fold. Subsequently, 100 μL of the well-mixed suspension was evenly plated onto lysogeny broth agar plates, and viable colonies were enumerated following 24 h of incubation. The bacterial pellets were harvested by centrifugation, rinsed three times with PBS and then fixed in 2.5% (v/v) glutaraldehyde solution. Following this, dehydration and drying were performed using an ethanol gradient, and the samples were, then, dehydrated, critical point dried, sputter-coated with gold and visualized using scanning electron microscopy (SEM). Bacterial survival area (%) was quantified via image analysis and calculated according to Equation (3):


(3) $$\text{Survival}\;\text{area}\;(\%)=S_{t}/S_{0}\times 100\%,$$

where *S_t_* is the colony area after co-culture with the test sample, and *S*_0_ denotes the colony area of the untreated control on agar plates.

### Cellular ROS-scavenging capacity of 3D-NEBS against H_2_O_2_-induced oxidative stress

To assess the ROS-scavenging activity of 3D-NEBS against H_2_O_2_-induced oxidative stress, human umbilical vein endothelial cells (HUVECs) were seeded in confocal microscopy culture dishes and cultured for 24 h. Cells were then treated with H_2_O_2_ (100 μM) and various hydrogels for another 24 h. Next, DCFH-DA (5 μM) (Beyotime, Shanghai) was added and incubated in the dark for 1 h, followed by the addition of DAPI and incubation for 20 min. ROS levels were quantified by measuring DCF fluorescence intensity using ImageJ after confocal imaging.

### Cell migration assay

The Transwell migration assay was used to test the recruitment performance of 3D-NEBS. Sterilized 3D-NEBS was placed at the bottom of a 24-well plate, and HUVECs were seeded in Transwell chambers at a density of approximately 2 × 10^5^ cells/well. Culture medium containing 2% FBS was added to both the upper and lower chambers to minimize FBS-mediated effects on cells.

### Wound scratch assay

HUVECs were seeded in 6-well plates and cultured until reaching confluence completely. A sterile pipette tip was used to create a scratch wound. The culture medium was replaced with DMEM containing 1% FBS, followed by co-culturing HUVECs with various hydrogels. Microscopic images of the scratch area were captured at 0 and 24 h using a Zeiss microscope, and quantitative analysis was performed using ImageJ software.

### Tube formation assay

Add 100 µL of Matrigel to each well of a 96-well plate, and incubate at 37°C for 1 h to induce gelation. Subsequently, seed HUVECs onto the gelled Matrigel and co-incubate with hydrogels for 6 h. Capture bright-field images of tube formation and quantify tubule number using ImageJ.

### Phenotype assessment of macrophage polarization

The hydrogel patches were placed in the 24-well plate Transwell chambers, with each well seeded with 2 × 10^5^ RAW264.7 cells for co-culture. Lipopolysaccharide and interleukin-4 (LPS 200 ng/mL, IL-4 50 ng/mL) were added to the culture medium to stimulate macrophage polarization into M1 and M2 phenotypes for 24 h, simulating the inflammatory microenvironment of diabetic infectious wounds. After 7 days of culture, the expression of pro-inflammatory M1 polarization-related genes IL-6 and TNF-α, as well as anti-inflammatory M2 polarization-related genes TGF-β1 and IL-10, was detected by ELISA. The expression of iNOS and Arg-1 in RAW264.7 cells was detected via immunofluorescence assay and observed using a Zeiss laser scanning confocal microscope. Subsequently, the hydrogel patches were placed in a 6-well plate, with each well seeded with 1.5 × 10^5^ Raw264.7 cells. The culture method was consistent with the aforementioned protocol, and materials were co-cultured with RAW264.7 cells for 7 days, and CD206 and CD86 were detected by immunofluorescence, followed by cell counting using flow cytometry.

### *In vitro* hemolysis assay

The experimental SD rats were procured from the Laboratory Animal Center of Nantong University, and all animal experiments were conducted in accordance with the protocol approved by the Institutional Animal Care and Use Committee of Nantong University (S20250225-001). All animal procedures, including surgery and postoperative management, were conducted in strict accordance with the Care and Use of Laboratory Animals. Briefly, whole blood was collected from healthy SD rats into sodium heparin tubes. Erythrocytes were isolated, resuspended in PBS and washed once with 1 mL PBS; supernatants were collected at set time points and absorbance measured at 540 nm. Lyophilized samples or Hydrosorb^®^ were dissolved in PBS to 1, 2, 4 and 8 mg/L. Each 500 μL sample solution was mixed with 500 μL erythrocyte suspension and incubated at 37°C with orbital shaking (150 rpm) for 1 h. After centrifugation (116 × *g*, 10 min), 100 μL supernatant was transferred to a microplate and read at 540 nm. 0.1% Triton X-100 and PBS served as positive and negative controls, respectively. Hemolysis rate was calculated using Equation (4):


(4) Hemolysis rate (%)=(Bs−Bp)/(Bw−Bp)×100%,

where Bs represents the absorbance at 540 nm of the sample suspension, Bp denotes that of PBS, and Bw corresponds to deionized water.

### *In vivo* rat hemostasis assay

All animal experiments were approved by the Nantong University Animal Ethics and Scientific Committee (Approval No.: S20250225-001). The experimental procedures strictly adhered to the ARRIVE (Animal Research: Reporting In Vivo Experiments) guidelines and were conducted in compliance with the United Kingdom’s Animals (Scientific Procedures) Act 1986 and its associated regulatory standards. Male SD rats were used to evaluate the hemostatic ability of 3D-NEBS. All SD rats were anesthetized with isoflurane and fixed on a wooden board for surgery. For the tail hemostasis experiment, one-third of the tail was cut off with surgical scissors, and a pre-weighed filter paper was placed under the wound. The wound was then exposed to the air for 15 s to allow free bleeding. Subsequently, the wound was covered with a 1 cm diameter circular 3D-NEBS, 3D-BS and Hydrosorb^®^ or left untreated. The hemostasis endpoint was defined as no further expansion of the bloodstain on the gauze and no blood seepage from the contact surface of 3D-NEBS gel, 3D-BS gel and Hydrosorb^®^ with the wound. Blood absorbed by pre-weighed filter paper was quantified. For liver hemostasis, the thoracic cavity was opened to expose the liver; a 0.5 cm³ wound was created with a biopsy needle, and 3D-NEBS was inserted into the puncture site. Blood loss was measured after 15 s (*n* = 6).

### Animal models and treatments

All animal experiments were approved by the Ethics and Scientific Committee for Experimental Animals at Nantong University (Approval No.: S20250225-001). The experimental procedures strictly adhered to the ARRIVE (Animal Research: Reporting In Vivo Experiments) guidelines and were conducted in compliance with the United Kingdom’s Animals (Scientific Procedures) Act 1986 and its associated regulatory standards. A total of forty 8-week-old male Sprague-Dawley (SD) rats, with a body weight of 200 ± 30 g, were utilized to establish an *in vivo* model of full-thickness diabetic wound healing. Diabetes was induced by a single intraperitoneal injection of streptozotocin (STZ, 45 mg/kg) dissolved in 0.1 M citrate buffer (pH 4.5). Four weeks later, rats with non-fasting blood glucose ≥16.7 mmol/L were selected for the study. Anesthesia was induced with isoflurane until loss of consciousness. Each rat was anesthetized with isoflurane to lose consciousness. A full-thickness circular incision with a diameter of 10 mm was carefully made on the back skin of each animal to simulate the wound site. Hydrogels with the same diameter as the wound and the same storage modulus were prepared according to the above method. The rats were divided into four groups (*n* = 6): Control group, Hydrosorb^®^ group, 3D-BS group and 3D-NEBS group. Wound images were captured using a digital camera and the wound closure area was quantified using ImageJ. During the entire study period, all rats were fed standard rodent chow *ad libitum* throughout the study. Wound healing was evaluated by digital planimetry on Day 3, 7, 9 and 14 post-injuries. Wound closure (%) was calculated as (*A_n_*/*A*_0_) × 100%, where *A_n_* is the wound area on Day *n* and *A*_0_ is the initial wound area measured immediately after wounding.

### H&E staining and Masson staining

On Day 14, diabetic rats were euthanized by CO_2_ inhalation, and wound-edge skin samples were collected, fixed in 10% neutral-buffered formalin, paraffin-embedded and sectioned. Sections were stained with H&E and Masson’s trichrome to assess wound healing.

### Immunofluorescence staining

Day 14 tissues underwent immunofluorescence staining: after dewaxing, antigen retrieval and blocking, sections were incubated with primary antibodies against IL-6, IL-1β and CD31; secondary antibodies; and DAPI. Images were acquired by confocal microscopy, and fluorescence intensity was quantified using ImageJ.

### *In vivo* compatibility

At week 4, blood was collected from rats in the 3D-NEBS group for serum biochemical analysis. The rats’ major organs were harvested, including liver, heart, spleen, kidneys and lungs and subjected to H&E staining to evaluate systemic histopathological safety and biocompatibility.

### Transcriptomic analysis of regenerated wound tissues

Transcriptome analysis of diabetic skin tissues from the control and 3D-NEBS groups was performed by Hangzhou Lianchuan Biotechnology Co., Ltd. Total RNA was extracted using TRIzol, quality-controlled and enriched for poly(A)+ mRNA. The mRNA was fragmented, reverse-transcribed into cDNA and converted to double-stranded DNA. End repair, A-tailing and adapter ligation were sequentially performed; the second strand was selectively degraded with UDG, followed by PCR amplification to generate a 300 ± 50 bp library. Sequencing was conducted on an Illumina NovaSeq 6000 platform. Differential gene expression analysis, Gene Ontology (GO) enrichment analysis and Kyoto Encyclopedia of Genes and Genomes (KEGG) pathway enrichment analysis were performed.

### Statistical analysis

All experiments were performed in triplicate or more. Quantitative data were analyzed using ImageJ and GraphPad Prism 9, and are presented as mean ± standard deviation (SD). Statistical significance was assessed by one-way analysis of variance (one-way ANOVA), followed by Tukey’s *post hoc* test for multiple comparisons. Differences were considered statistically significant at *P < 0.05, **P < 0.01, ***P < 0.001 and ****P < 0.0001; ns denotes not significant.

## Results and discussions

### Structural characterization of the PGL nanoparticles and bio-ink matrix

**Figure 1:**
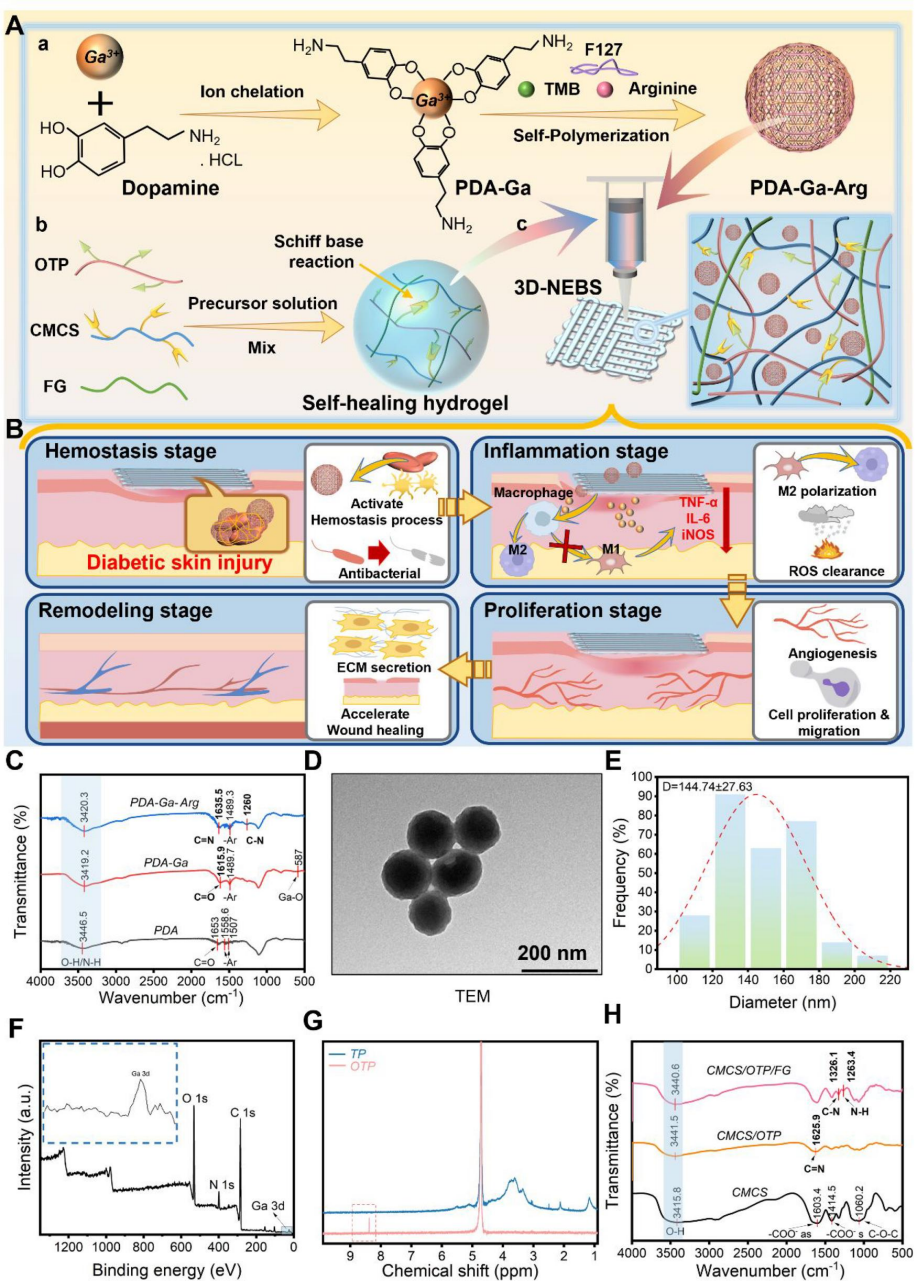
Schematic illustration of the 3D-NEBS fabrication process and underlying mechanism. (**A**) Hierarchical fabrication process: (**a**) synthesis of polydopamine-gallium-arginine nanoparticle (PDA-Ga-Arg) via metal-phenolic coordination, (**b**) formation of a dynamic hydrogel network through Schiff base crosslinking between oxidized *Tremella* polysaccharide (OTP), carboxymethyl chitosan (CMCS) and fish gelatin (FG) and (c) 3D bioprinting to generate functional wound dressings. (**B**) The proposed therapeutic mechanisms of 3D-NEBS in coordinating the four phases of diabetic wound healing. (**C**) FTIR spectra confirming the successful coordination between Ga³^+^ and the phenolic hydroxyl/quinone groups of PDA. (**D**) TEM image revealing the spherical morphology of the PDA-Ga-Arg nanoparticles. (**E**) Size distribution histogram indicating good monodispersity. (**F**) XPS spectra providing evidence for the chemical state of Ga and its coordination with N and O atoms. (**G**) ^1^H NMR spectra verifying the successful oxidation of *Tremella* polysaccharide (TP) to OTP, as indicated by the emerging aldehyde proton peak. (**H**) FTIR spectra demonstrating the successful formation of imine bonds (C=N) upon hydrogel crosslinking.

To address the pathologically altered microenvironment of diabetic wounds, we designed and synthesized a multifunctional polydopamine–gallium–arginine nanoparticle (denoted as PGL) via a metal–catechol coordination strategy [36]. As illustrated in Figure 1A, the synthesis initiates under alkaline conditions where dopamine chelates Ga³^+^ to form a PDA-Ga intermediate [37]. Subsequent interfacial polymerization in the presence of L-arginine, using 1,3,5-trimethylbenzene as a soft template and Pluronic F127 as a surfactant, yielded monodisperse spherical nanoparticles 144 ± 27 nm (Figure 1D and E). The uniform morphology and nanoscale size of PGL are advantageous for biomedical applications due to enhanced bioavailability and cellular uptake.

The successful synthesis and functionalization of PGL were confirmed through a suite of spectroscopic techniques. FTIR analysis (Figure 1C) revealed a redshift in the C=O stretching vibration shifted from 1653 cm^−1^ in pure PDA to 1616 cm^−1^ in PDA–Ga, indicative of successful coordination between Ga³^+^ and quinone groups. Additional peaks emerged at 1636 cm^−1^ (bande d’étirement C=N) and 1260 cm^−1^ (bande d’étirement C–N), accompanied by a broadened O–H/N–H band around 3420 cm^−1^, confirming the incorporation of L-arginine and participation in hydrogen bonding. XPS analysis (Figure 1F) provided further evidence, with Ga 3d peaks observed at 20.5 eV and 22.1 eV, corresponding to Ga–O and potential Ga–N coordination, respectively. EDS elemental mapping (Supplementary Figures S3 and S15) demonstrated a uniform spatial distribution of Ga throughout the nanoparticles, affirming successful integration into the PDA matrix.

The uniform dispersion and integration of the PDA-Ga-Arg nanoparticles within the 3D-NEBS hydrogel network were verified using complementary techniques. Due to the nanoscale size of the particles and the similar electron contrast between their polydopamine-based composition and the organic hydrogel matrix, individual nanoparticles are not distinctly resolved in conventional SEM images. To directly assess their distribution, high-resolution Cryo-fractured hydrogel sections were subjected to SEM imaging coupled with EDS mapping. The corresponding Ga elemental maps revealed a homogeneous spatial distribution without aggregation or localized enrichment, confirming the macroscopic uniformity of nanoparticle incorporation throughout the scaffold (Supplementary Figure S14).

For the hydrogel scaffold, *Tremella* polysaccharide (TP) was selectively oxidized to OTP to introduce aldehyde functionalities, enabling dynamic Schiff base cross-linking. The successful oxidation was verified via ^1^H NMR spectroscopy (Figure 1G), which displayed a characteristic aldehyde proton signal at approximately 8.2 ppm. The hydrogel network was formed through a Schiff base reaction between OTP, carboxymethyl chitosan (CMCS) and fish gelatin (FG), as corroborated by the appearance of an imine bond peak at 1626 cm^−1^ in the FTIR spectrum (Figure 1H). Peaks corresponding to the amide III bands of FG at 1326 and 1263 cm^−1^ further indicated the formation of a multi-network hydrogel stabilized by both covalent cross-linking and hydrogen bonding.

The concentration of PDA-Ga-Arg nanoparticles within the 3D-NEBS hydrogel was quantitatively determined based on the initial feeding ratio and subsequent purification yield. After synthesis and lyophilization, the PDA-Ga-Arg nanoparticles were uniformly dispersed into the COF (CMCS/OTP/FG) precursor solution (5.0 mg/mL). This corresponded to a final nanoparticle content of approximately 0.5 wt% in the crosslinked hydrogel (assuming a total solid content of ∼10 wt% for the hydrogel matrix). This concentration was optimized through preliminary dose-response studies, which balanced the hydrogel’s printability, mechanical robustness, ROS-scavenging capacity and immunomodulatory activity without compromising cytocompatibility.

Given the critical importance of gallium ion (Ga³^+^) biosafety, we further quantified its cumulative release profile from 3D-NEBS under simulated wound conditions (pH 5.5, 6.5 and 7.4). Based on the predetermined PDA-Ga-Arg nanoparticle loading (1.0 wt% in the hydrogel, with Ga accounting for ≈10 wt% of the nanoparticles), cumulative Ga³^+^ release was quantified by ICP-MS. Release kinetics were pH-responsive and sustained. At pH 5.5, approximately 80% of the loaded Ga³^+^ was released over 10 days, corresponding to a total release of less than 800 μg per gram of dried hydrogel (Supplementary Figure S11).

To assess potential systemic exposure, the released Ga³^+^ amount was translated into an equivalent *in vivo* dose. Assuming full transdermal absorption from a single 3D-NEBS dressing (≈200 mg dry weight) applied to a standard Sprague-Dawley rat (≈250 g), the maximum equivalent systemic dose of Ga³^+^ would be approximately 0.4 mg kg^−1^. This value is well below the reported toxicity threshold for gallium in rodents (≈4 mg kg^−1^), indicating a substantial safety margin. These *in vitro* release data, together with our comprehensive *in vivo* biosafety evaluations (hematology, serum biochemistry and histopathology of major organs), collectively confirm that Ga³^+^ release from 3D-NEBS is controlled and poses negligible systemic risk.

**Figure 2:**
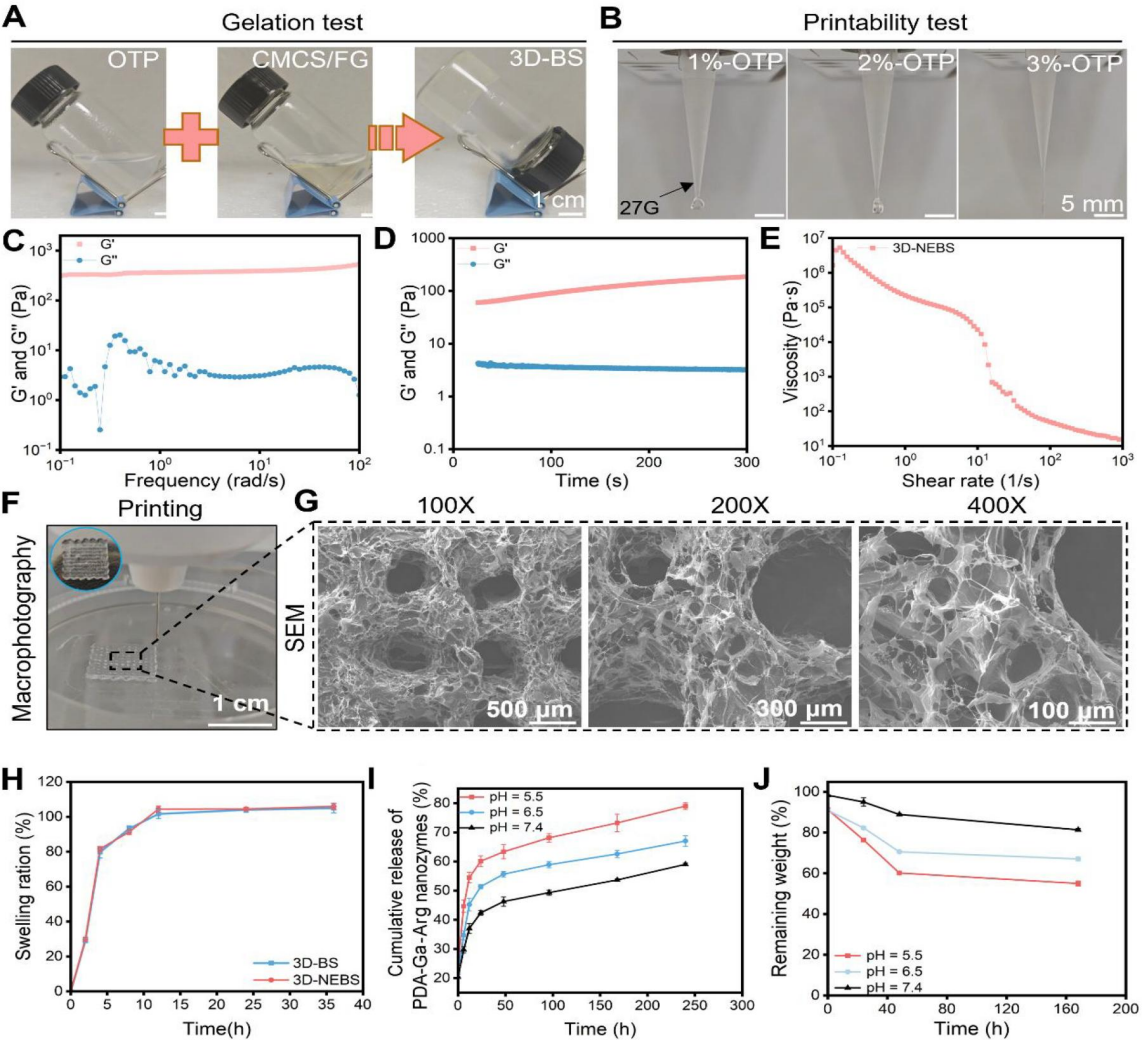
Rheological properties, printability and responsive behaviors of the hydrogels. (**A**) Photographs showing the rapid gelation process. (**B**) A representative image of the precise 3D printing process. (**C**, **D**) Rheological measurements showing the viscoelastic properties (*G*′ > *G*″) and (**E**) pronounced shear-thinning behavior, (**F**) essential for extrusion-based 3D printing. (**G**) SEM images acquired at multiple magnifications reveal the uniform, interconnected porous microstructure of the lyophilized hydrogel. (**H**) Swelling profiles of 3D-BS and 3D-NEBS in PBS (*n* = 3). (**I**) pH-responsive release kinetics of PDA-Ga-Arg nanoparticles under simulated wound environments (pH 5.5, 6.5, 7.4; *n* = 3). (**J**) Degradation profiles under different pH conditions (*n* = 3).

The optimized hydrogel precursor, comprising 3% OTP, 5% CMCS and fish gelatin, demonstrated rapid crosslinking (Figure 2A) and exceptional self-healing capability (Supplementary Figure S4), essential for handling mechanical stress during application. The hydrogel synthesis schematic is shown in Supplementary Figure S2. The formulation exhibited outstanding printability, allowing precise fabrication of complex structures via extrusion-based 3D printing (Figure 2B and Supplementary Video S1 and Table S1). Rheological characterization confirmed robust viscoelastic properties, with the *G*′ consistently *G*″ across measured frequencies (Figure 2C and D). Notably, the hydrogel displayed pronounced shear-thinning behavior (Figure 2E), facilitating smooth extrusion and shape retention post-printing, which is critical for constructing tailored wound dressings.

Utilizing extrusion-based 3D printing, the hydrogel was accurately patterned into uniform, patch-like architectures (Figure 2F). SEM revealed a highly porous and interconnected microstructure within the 3D-BS scaffold (Figure 2G and Supplementary Figure S17), promoting exudate absorption, oxygen permeation and tissue integration—attributes vital for diabetic wound management. Swelling kinetics experiments indicated that both 3D-BS and nanoparticle enhanced 3D-NEBS reached absorption equilibrium within 12 h (Figure 2H), with the nanoparticles-loaded group showing marginally higher swelling capacity, likely due to the hydrophilic nature of the embedded PGL nanoparticles.

A defining feature of the 3D-NEBS system is its intelligent, pH-responsive release. At pH 5.5, simulating the diabetic wound microenvironment, the release of PGL nanoparticles was significantly accelerated compared to neutral pH (7.4), achieving nearly 80% release within 72 h without an initial burst effect (Figure 2I). This controlled release is attributed to the acid-labile Schiff base linkages and slightly swollen hydrogel matrix under acidic conditions, ensuring targeted therapeutic delivery where most needed. Consistent with this release kinetics, degradation studies showed faster dissolution of the hydrogel under acidic pH, while it remained stable at physiological pH (Figure 2J), underscoring its environmental sensitivity and suitability for long-term application.

Given the pivotal role of vascular regeneration during diabetic wound healing, we evaluated the cytocompatibility of the PGL nanoparticles using HUVECs. The CCK-8 assay revealed no significant cytotoxicity up to 500 μg/mL (Supplementary Figure S5), demonstrating high biocompatibility.

In summary, the 3D-NEBS platform integrates several advanced functionalities into a single tissue-engineered construct: exceptional printability for personalized dressing design, self-healing capability for durability, pH-responsive release for intelligent drug delivery, porous microstructure for tissue support. This multifaceted system advances the development of high-performance bionic skin substitutes for diabetic wound treatment.

### 3D-NEBS reprograms macrophage polarization and alleviates inflammation

**Figure 3:**
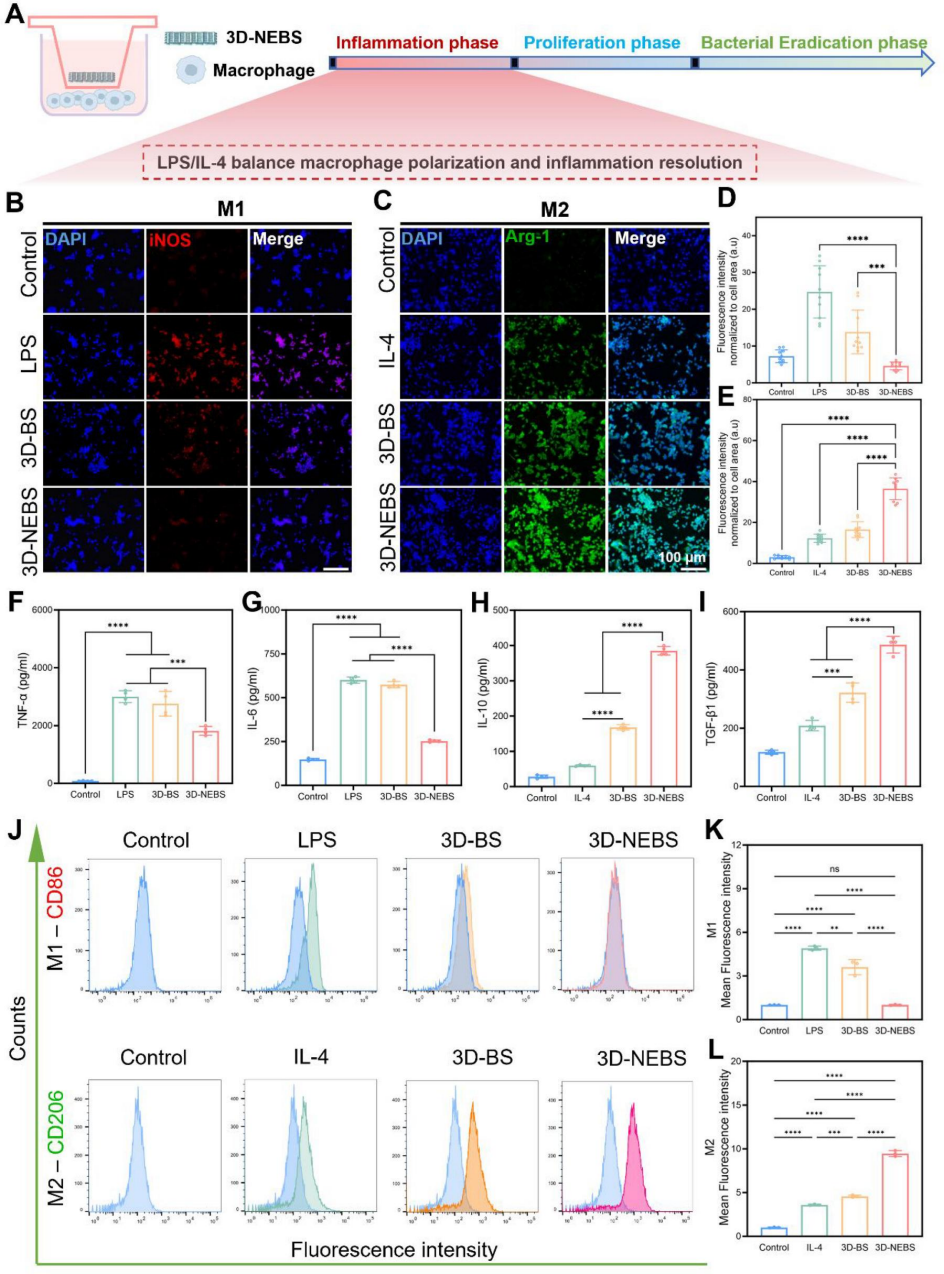
Immunomodulatory reprogramming of macrophages by 3D-NEBS via phenotypic switching from M1 to M2. (**A**) Schematic illustration of the macrophage–3D-NEBS coculture system under inflammatory stimulation. (**B**) Representative immunofluorescence images of iNOS in LPS-stimulated macrophages. (**C**) Representative immunofluorescence images of Arg-1 in IL-4-stimulated macrophages. (**D**, **E**) Quantitative analysis of iNOS and Arg-1 fluorescence intensity normalized to cell area (*n* = 10). (**F**–**I**) ELISA quantification TNF-α, IL-6, IL-10 and TGF-β1 cytokine secretion levels (*n* = 4). (**J**–**L**) Flow cytometry analysis and quantification of CD86^+^ and CD206^+^ macrophage populations (*n* = 3). (ns: not significant, *P < 0.05, **P < 0.01, ***P < 0.001, ****P < 0.0001).

Chronic diabetic wounds are characterized by a prolonged inflammatory microenvironment, where dysregulated macrophage polarization—specifically, the dominance of pro-inflammatory M1 over pro-healing M2 phenotypes—significantly impedes the healing process. To evaluate the immunomodulatory potential of 3D-NEBS, we employed an *in vitro* RAW264.7 macrophages model stimulated with interleukin-4 (IL-4) to promote M2 polarization, and lipopolysaccharide (LPS) to drive M1 polarization, as illustrated in Figure 3A. A control hydrogel without nanoparticles (3D-BS) was used for comparison. Prior to immunomodulation assays, biocompatibility was demonstrated via live/dead staining and CCK-8 assays, which revealed high cell viability in both macrophages and HUVECs treated with 3D-NEBS and 3D-BS (Supplementary Figures S6 and S7), underscoring the excellent cytocompatibility of the hydrogel system.

Notably, macrophages treated with 3D-NEBS under LPS and IL-4 stimulation exhibited a pronounced shift in polarization state. Immunofluorescence analysis demonstrated a significant reduction in iNOS, and increase in Arg-1 expression (Figure 3B–E). This effect was more substantial than that achieved with IL-4 stimulation alone, highlighting the synergistic role of the hydrogel in driving macrophage repolarization. Consistent with these observations, enzyme-linked immunosorbent assay (ELISA) confirmed a substantial reduction in pro-inflammatory cytokines–specifically TNF-α and IL-6 and a concomitant increase in anti-inflammatory mediators–namely IL-10 and TGF-β1 in the 3D-NEBS group (Figure 3F–I).

Further validation using flow cytometry confirmed a significant shift in macrophage polarization: CD86 (M1 marker) expression decreased, while CD206 (M2 marker) expression increased in both the 3D-BS and 3D-NEBS groups, with the latter exhibiting superior immunomodulatory efficacy (Figure 3J–L). These results collectively demonstrate that 3D-NEBS effectively reprograms macrophage polarization from a pro-inflammatory toward a pro-regenerative phenotype, thereby mitigating chronic inflammation and fostering a healing-conducive microenvironment. The enhanced immunomodulatory efficacy stems from the synergistic effects of the antioxidant *Tremella* polysaccharide matrix and the multifunctional PDA-Ga-Arg nanoparticles, which collectively scavenge ROS and facilitate metabolic reprogramming of immune cells.

This robust M1-to-M2 transition induced by 3D-NEBS underscores its therapeutic potential as an immunomodulatory wound dressing for diabetic wound, aligning with the critical need to resolve inflammation in the early phases of wound healing.

### 3D-NEBS promotes angiogenesis via ROS scavenging and NO-mediated signaling in a diabetic microenvironment

**Figure 4:**
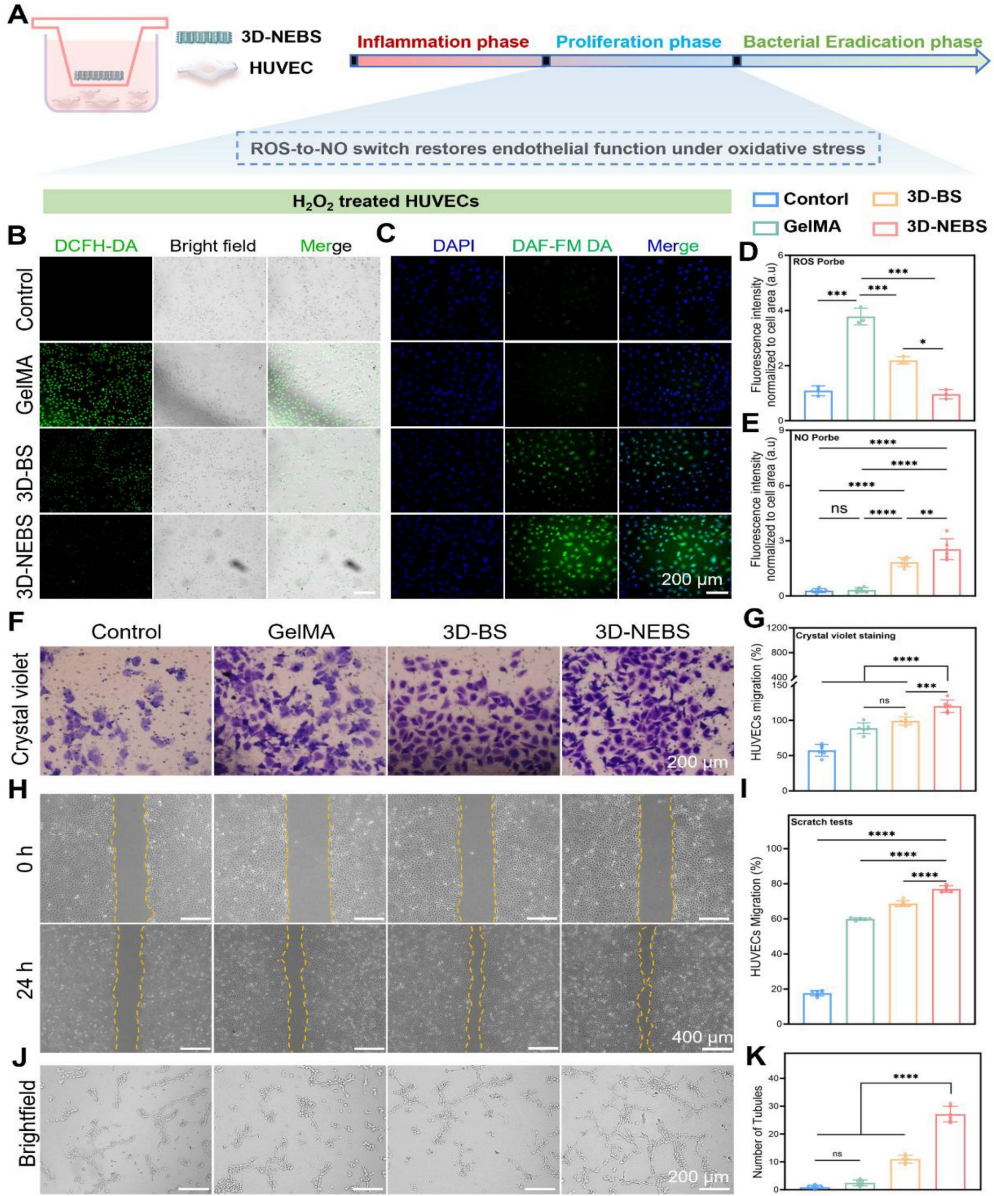
3D-NEBS rescues endothelial function under oxidative stress by modulating ROS–NO signaling and promoting angiogenesis. (**A**) Schematic of the experimental setup for the H_2_O_2_-induced oxidative stress model and 3D-NEBS treatment in HUVECs. (**B**, **C**) Representative confocal microscopy images of intracellular (**B**) ROS levels (stained with DCFH-DA) and (**C**) NO production (stained with DAF-FM); (**D**, **E**) quantitative analysis of (**D**) ROS and (**E**) NO, respectively (*n* = 3 for ROS, *n* = 6 for NO). (**F**, **G**) Transwell migration assay: (**F**) representative images of crystal violet-stained migrated cells and (**G**) quantitative analysis (*n* = 6). (**H**, **I**) Wound healing scratch assay: (**H**) representative images at 0 and 24 h and (**I**) quantification of wound closure rate (*n* = 6). (**J**, **K**) Tube formation assay: (**J**) representative images of capillary-like structures formed on Matrigel and (**K**) quantification of branch points (*n* = 6). (ns: not significant, *P < 0.05, **P < 0.01, ***P < 0.001, ****P < 0.0001).

Oxidative stress, characterized by excessive accumulation of ROS, is a major impediment to angiogenesis in diabetic wounds [38–41]. To investigate the pro-angiogenic mechanism of 3D-NEBS, we employed an H_2_O_2_-induced oxidative damage model in HUVECs, focusing on the ROS–NO signaling axis (Figure 4A). Prior to cellular assays, the intrinsic antioxidant capacity of 3D-NEBS was confirmed by DPPH radical scavenging tests (Supplementary Figure S8), demonstrating excellent free radical elimination ability. Subsequent intracellular ROS detection using the DCFH-DA probe revealed that 3D-NEBS significantly reduced ROS levels in HUVECs under oxidative stress (Figure 4B and D). Concurrently, 3D-NEBS promoted nitric oxide (NO) generation via the arginine metabolic pathway (Figure 4C and E). This bidirectional regulation—scavenging excess ROS while enhancing NO production—collectively reshaped the hostile oxidative microenvironment into one conducive to vascular regeneration.

Functional assays consistently demonstrated the pro-angiogenic effects of 3D-NEBS. Transwell migration assays revealed a significant increase in endothelial cell migration (Figure 4F and G). Scratch wound healing experiments further confirmed enhanced directional cell movement (Figure 4H and I). Most notably, tube formation assays showed that HUVECs treated with 3D-NEBS developed more extensive and mature vascular-like networks, with increased branching points and total tube length compared to controls (Figure 4J and K).

The beneficial effects are attributed to the rational design and structural integration of endogenous bioactive components: (i) the inherent ROS-scavenging capacity of the functional hydrogel matrix alleviates oxidative damage and protects endothelial function; (ii) the sustained release of NO facilitates vasodilation and activates the VEGF signaling pathway, a master regulator of angiogenesis; and (iii) the *Tremella* polysaccharide-based hydrogel provides a biomimetic 3D microenvironment that supports cell adhesion, spreading and phenotypic maturation. Rather than relying on external catalysts, our innovation lies in engineering an “endogenous” therapeutic system that functions as an intelligent, multifunctional wound microenvironment “regulator.” This multi-targeted approach—simultaneously mitigating oxidative stress, promoting vasodilatory signaling and providing mechanical support—overcomes the limitations of traditional strategies dependent solely on growth factor delivery, offering a comprehensive solution for vascular regeneration in diabetic wounds.

### Evaluation of the broad-spectrum antibacterial properties of 3D-NEBS

**Figure 5:**
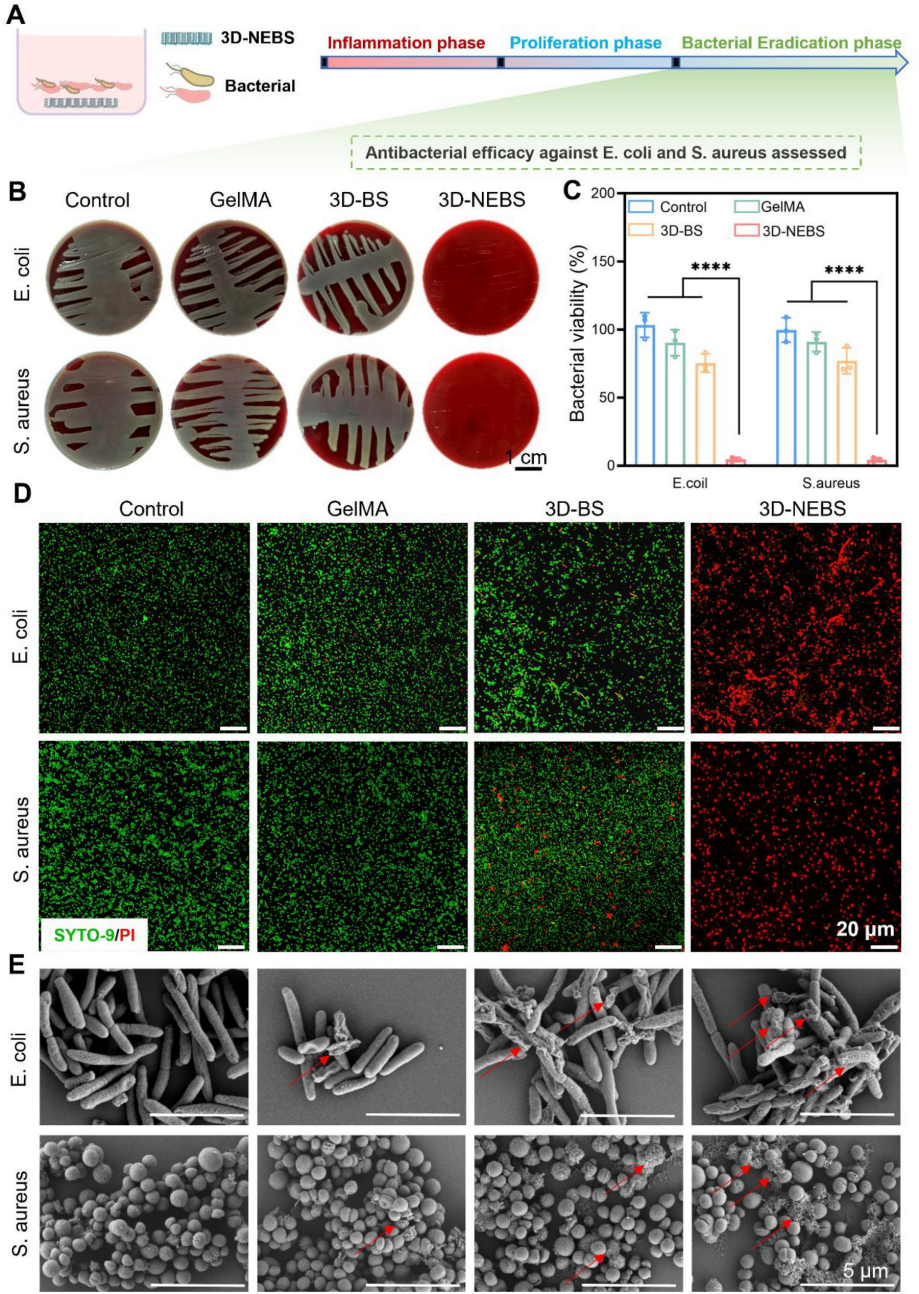
3D-NEBS exhibits broad-spectrum antibacterial activity against diabetic wound-relevant pathogens through multimodal mechanisms. (**A**) Schematic illustration of the experimental workflow for evaluating antibacterial performance. (**B**) Representative images of bacterial colony formation on agar plates after various treatments. (**C**) Quantitative analysis of bacterial survival rates (*n* = 3). (**D**) Live/dead fluorescence staining of bacteria (green: viable; red: membrane-compromised/dead). (**E**) Representative SEM images showing morphological damage to bacterial membranes; red arrows indicate sites of membrane disruption. (****P < 0.0001).

Bacterial infection, particularly polymicrobial infections involving *S. aureus* and *E. coli*, significantly delays diabetic wound healing by exacerbating inflammation and impairing tissue regeneration [42–44]. To evaluate the antibacterial properties of 3D-NEBS, we performed systematic *in vitro* assays against these clinically relevant strains (Figure 5A). The results indicated potent and broad-spectrum antibacterial activity. Plate counting assays revealed a significant reduction in bacterial viability in the 3D-NEBS group relative to all control groups (Control, GelMA and 3D-BS), with highly significant differences (****P < 0.0001; Figure 5B and C). Live/dead staining further confirmed extensive bacterial death, indicating severe membrane disruption (Figure 5D). Scanning electron microscopy (SEM) images visually corroborated these findings, showing clear morphological damage such as membrane rupture and cytoplasmic leakage in both Gram-positive and Gram-negative bacteria species (Figure 5E).

Given that diabetic wounds are frequently complicated by bacterial biofilms, we further evaluated the ability of 3D-NEBS to inhibit biofilm formation using an *in vitro* model. Biofilm biomass of *S. aureus* and *E. coli* was quantified via crystal violet staining. As shown in Supplementary Figure S12, 3D-NEBS significantly suppressed early-stage biofilm formation compared to control, GelMA, and 3D-BS groups, reducing biofilm biomass by approximately 75–95%. This robust anti-biofilm efficacy aligns with its potent activity against planktonic bacteria and can be attributed to a multi-modal action: (i) Ga³^+^ ions interfere with bacterial iron metabolism, potentially disrupting quorum sensing and exopolysaccharide (EPS) production essential for biofilm stability; (ii) the polydopamine (PDA) component adheres to nascent biofilm structures, promoting physical disruption; and (iii) the pH-responsive release of nanoparticles in the acidic wound microenvironment ensures targeted delivery of these active agents to the biofilm site. These results demonstrate that 3D-NEBS is effective not only against free-living bacteria but also against the structured, adherent biofilm communities commonly encountered in chronic diabetic wounds.

The antibacterial mechanism of 3D-NEBS can be attributed to a multi-modal strategy: (i) Ion Interference: Ga³^+^ ions competitively inhibit bacterial iron uptake, disrupting respiratory enzyme activity and metabolic function, and further impair biofilm formation by interfering with quorum sensing and exopolysaccharide production; (ii) Membrane Targeting: Polydopamine (PDA) facilitates adhesion to both planktonic bacteria and nascent biofilm structures, inducing physical membrane disruption and biofilm destabilization; (iii) Physical Barrier Effect: The hydrogel’s nanoporous structure limits bacterial penetration and mitigates biofilm formation. Notably, the combination of these mechanisms reduces the risk of antibiotic resistance development—a critical advantage over conventional treatments. The potent anti-biofilm activity, as confirmed in Supplementary Figure S12, further underscores the potential of 3D-NEBS as an effective antibacterial dressing for managing infected diabetic wounds, which are often plagued by resilient biofilm communities. Notably, this multimodal mechanism mitigates antibiotic resistance development—a critical advantage over conventional antimicrobial therapies. These findings demonstrate that 3D-NEBS functions as an effective antibacterial dressing for infected diabetic wounds.

### Multimodal diabetic wound therapy based on excellent hemocompatibility and hemostatic performance: from microenvironment regulation to tissue regeneration

Prior to evaluating the *in vivo* wound healing efficacy of 3D-NEBS, we systematically assessed its hemocompatibility and hemostatic properties to ensure clinical safety and therapeutic potential. Hemolysis assays revealed that 3D-NEBS exhibited negligible hemolytic activity, with hemolysis rates below 5% (Supplementary Figure S9A and B), confirming its excellent blood compatibility without causing significant erythrocyte damage. Furthermore, *in vivo* hemostasis experiments using rat tail amputation and liver hemorrhage models demonstrated that 3D-NEBS significantly reduced blood loss and shortened clotting time compared to control groups (Supplementary Figure S9C–E), highlighting its superior hemostatic capability. These properties not only establish a stable and non-thrombogenic microenvironment conducive to diabetic wound healing but also mitigate early-stage bleeding and excessive inflammatory responses, thereby facilitating subsequent repair processes.

**Figure 6:**
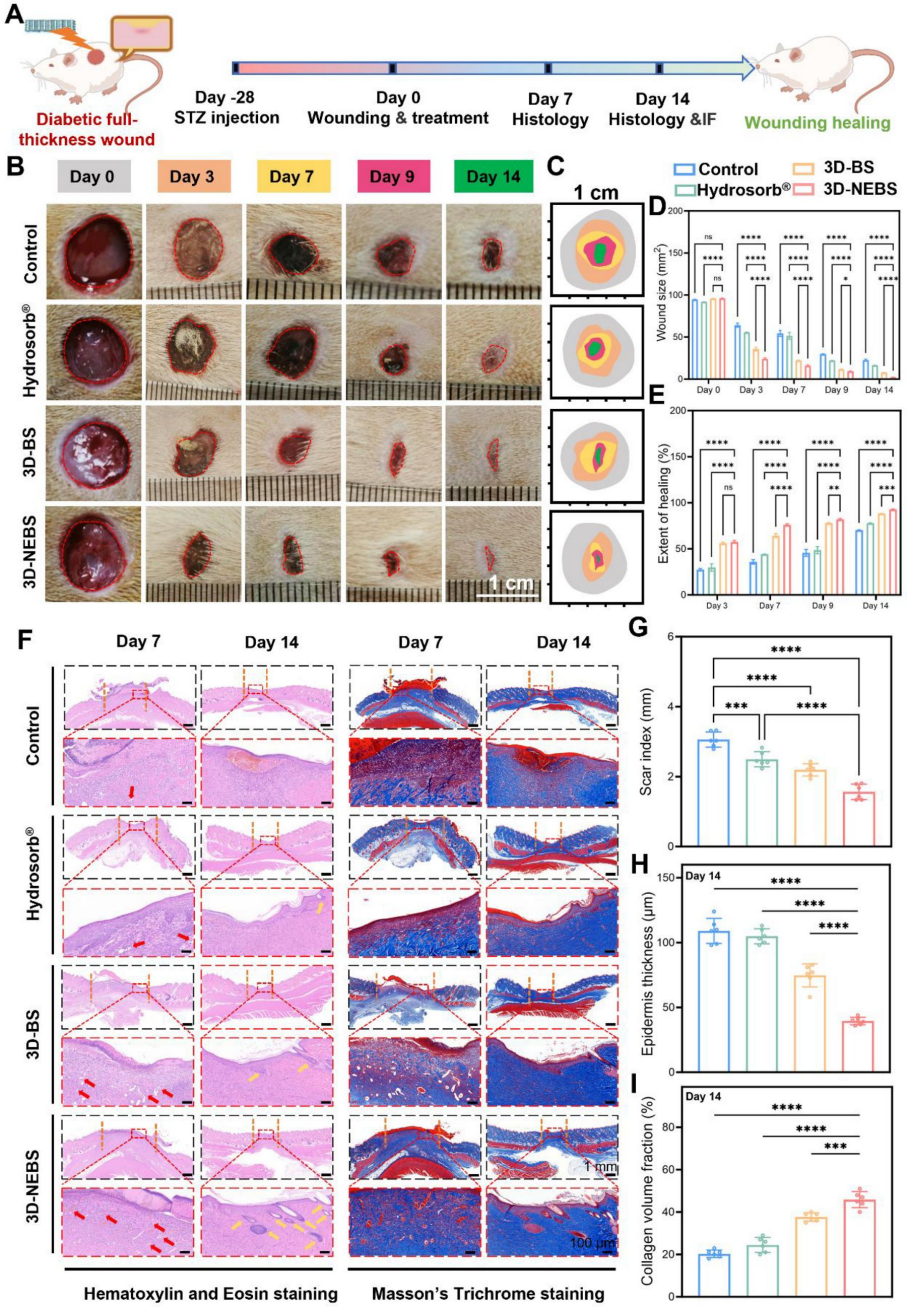
*In vivo* evaluation of the therapeutic efficacy of 3D-NEBS in diabetic wound healing. (**A**) Experimental timeline: STZ-induced diabetic rat model development (28 days), wound creation and treatment initiation (Day 0) and tissue collection (Day 7/14). (**B**) Representative macroscopic images of wound closure at Day 0, 3, 7, 9 and 14. (**C**) Time series diagram of wound area reduction. (**D**, **E**) Quantitative analysis of (**D**) wound size and (**E**) healing percentage (*n* = 6). (**F**) H&E and Masson’s trichrome staining of wound tissues at Day 7/14. (**G–I**) Histomorphometry quantification of (**G**) scar index, (**H**) epidermal thickness and (**I**) collagen volume fraction (*n* = 6). (ns: not significant, *P < 0.05, **P < 0.01, ***P < 0.001, ****P < 0.0001).

Building upon the comprehensive advantages of 3D-NEBS in antioxidative, anti-inflammatory, antibacterial and hemostatic performance, we employed diabetic rat model bearing full-thickness cutaneous wounds (10 mm in diameter) to systematically evaluate its wound healing efficacy (Figure 6A). Throughout the 14-day treatment period, the 3D-NEBS group exhibited markedly accelerated wound closure compared to other groups (Figure 6B). Quantitative analysis showed that the 3D-NEBS group exhibited a wound area reduction from 96.21 ± 0.15 mm^2^ to 42.41 ± 1.51 mm^2^ by Day 3, corresponding to a healing rate of 57.56% ± 1.5%, which further increased to 92.79% ± 0.39% by Day 14 (Figure 6C–E). These results underscore the rapid and sustained wound healing promoted by 3D-NEBS.

To comprehensively assess the therapeutic outcomes, regenerated wound tissues from all groups were harvested on Day 7 and 14 for detailed histopathological analysis, including H&E and Masson staining (Figure 6F). Histopathological evaluation assessed key wound-healing parameters, including the scar index, epidermal thickness and collagen deposition. The 3D-NEBS group displayed the lowest scar index among all groups, indicating its capacity to promote scar-minimized healing (Figure 6G). Moreover, the epidermal layer in the 3D-NEBS-treated wounds was significantly thinner, suggesting effective prevention of hypertrophic scar or keloid formation (Figure 6H). Masson’s staining further revealed that the 3D-NEBS group exhibited denser, more organized and mature collagen fibers with enhanced alignment, indicative of improved extracellular matrix (ECM) remodeling and tissue regeneration (Figure 6I). These histological findings collectively demonstrate that 3D-NEBS not only accelerates wound closure but also enhances the quality of healing by promoting structurally and functionally superior tissue regeneration.

The long-term biosafety of Ga³^+^ released from 3D-NEBS was rigorously evaluated. The initial Ga³^+^ load was minimized by design, with PDA-Ga-Arg nanoparticles incorporated at 1.0 wt% (Ga comprising ≈10 wt% of nanoparticles). Release kinetics under simulated wound conditions (pH 5.5) showed sustained, pH-responsive liberation of ≈80% of loaded Ga³^+^ over 10 days. Even assuming complete release, the calculated maximum systemic exposure dose remains substantially below the established toxicity threshold for rodents. Released Ga³^+^ is expected to follow a well-characterized metabolic pathway—binding to serum transferrin and undergoing renal excretion—which limits accumulation in vital organs. Supporting *in vivo* evidence from a 4-week study confirmed the absence of systemic toxicity: histopathology of major organs revealed normal architecture without inflammation, necrosis or fibrosis (Supplementary Figure S13) and blood biochemistry (hepatic/renal markers) and hematology profiles showed no significant deviations from controls, all within normal physiological ranges. Collectively, these data affirm that Ga³^+^ release from 3D-NEBS is controlled and poses negligible long-term biosafety risks within the experimental model.

To further assess the translational potential of 3D-NEBS, we conducted a comparative study evaluating its long-term stability, mechanical durability and functional retention against several widely-used clinical dressings (gauze, Alginate^®^ and Aquacel-Ag^®^). After immersion in simulated body fluid (SBF) for 4 weeks, 3D-NEBS maintained its structural integrity without noticeable disintegration, whereas some commercial dressings exhibited swelling or deformation (Supplementary Figure S16C). Mechanical testing revealed that the Young’s modulus of 3D-NEBS post-immersion remained significantly higher than that of all commercial counterparts, indicating superior mechanical robustness in a hydrated state (Supplementary Figure S16D and E). Importantly, SEM-EDS mapping of the soaked 3D-NEBS confirmed that Ga-signal remained uniformly distributed throughout the hydrogel matrix, demonstrating stable incorporation and dispersion of the nanoparticles without aggregation or leaching (Supplementary Figure S17). Furthermore, the anti-biofilm activity against *S. aureus* and *E. coli* was well preserved after the long-term soaking, with biofilm biomass reduction rates of approximately 70–90%, outperforming the control dressings (Supplementary Figure S16A and B). These data collectively highlight that 3D-NEBS not only offers multifunctional therapeutic actions but also exhibits exceptional long-term stability, mechanical resilience and sustained bioactive performance, positioning it as a durable and effective candidate for managing persistent diabetic wounds.

**Figure 7:**
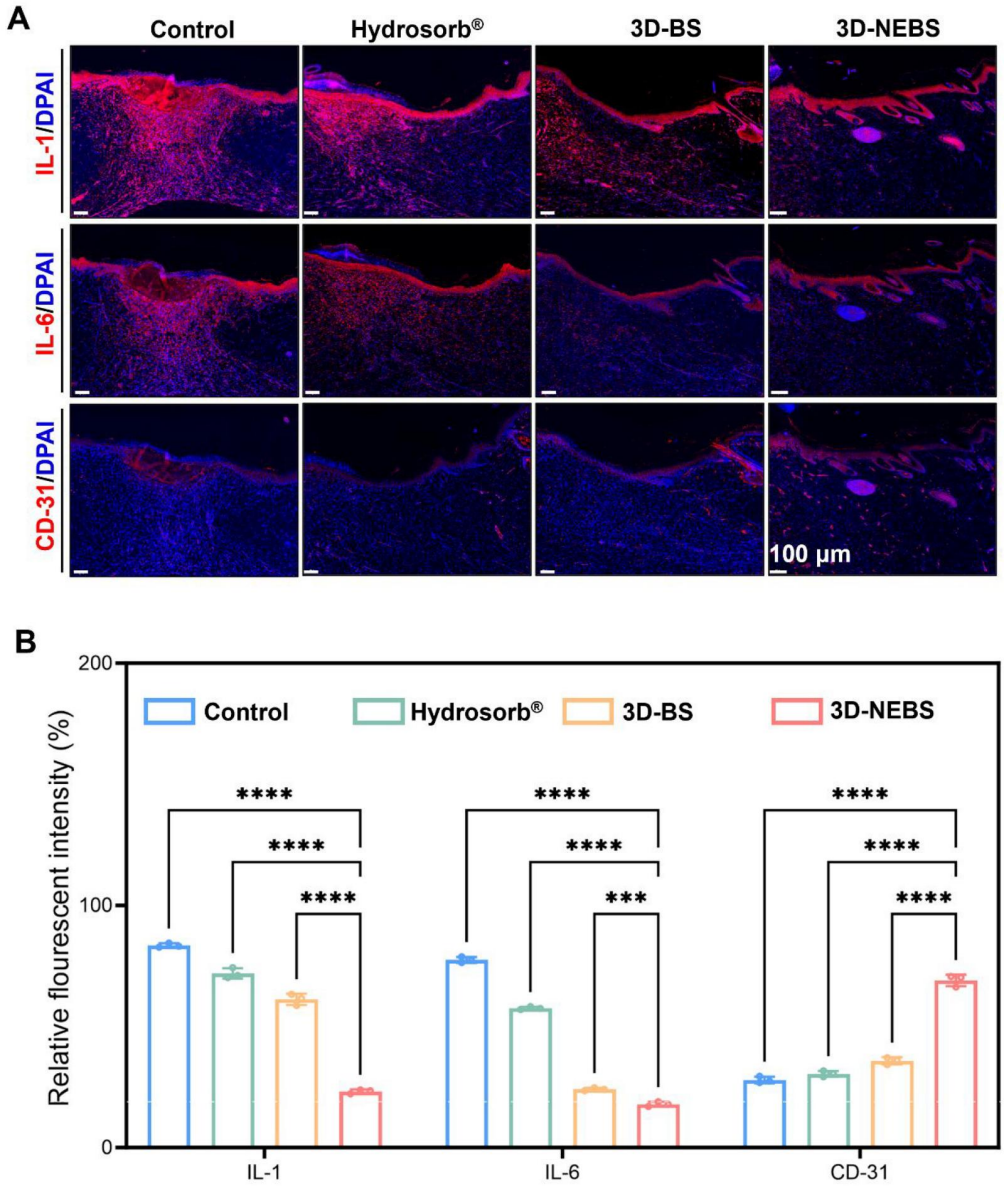
Immunomodulatory and pro-angiogenic effects of 3D-NEBS in diabetic wounds at Day 14. (**A**) Representative immunofluorescence staining of: Pro-inflammatory markers (IL-1, IL-6) vascular marker (CD31). (**B**) Quantitative analysis of: IL-1 and IL-6 expression levels (inflammatory response) CD31-positive area (neovascularization; *n* = 3). (***P < 0.001, ****P < 0.0001).

Immunofluorescence staining (Figure 7A) showed reduced expression of IL-1β and IL-6, but stronger expression of vascular marker CD31 in the 3D-NEBS group. Further quantitative analysis (Figure 7B) confirmed its ability to optimize the healing microenvironment through inflammation regulation and angiogenesis promotion. Safety evaluation through hematological tests (Supplementary Table S1) and histopathological examination of major organs (Supplementary Figure S10) showed no significant differences between 3D-NEBS-treated and control groups, demonstrating excellent biosafety. These results provide crucial experimental evidence for clinical translation of 3D-NEBS.

### Transcriptomic profiling reveals multimechanistic regulation of diabetic wound healing by 3D-NEBS

**Figure 8:**
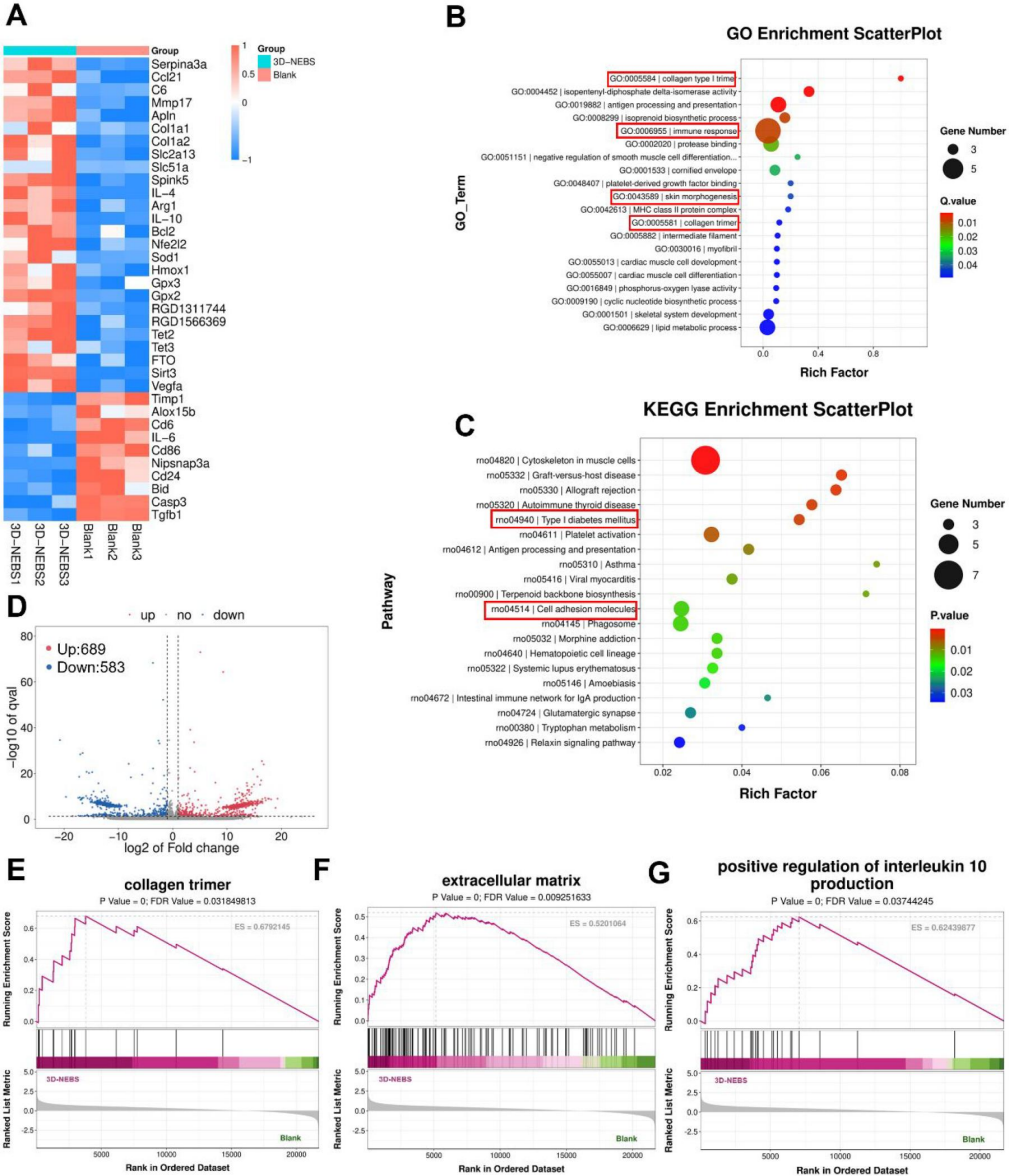
Transcriptomic profiling reveals molecular mechanisms of 3D-NEBS in diabetic wound healing. (**A**) Heatmap analysis of differentially expressed genes in the 3D-NEBS group versus the blank control group. (**B**) GO term enrichment analysis. (**C**) KEGG pathway enrichment analysis. (**D**). Volcano plot comparing differentially expressed genes between the control and 3D-NEBS groups. Upregulated genes are indicated in red; downregulated genes, in blue. (**E**–**G**) Gene Set Enrichment Analysis (GSEA) of: collagen trimer assembly, extracellular matrix organization and positive regulation of interleukin-10 production.

To systematically decipher the molecular mechanisms by which 3D-NEBS promotes diabetic wound regeneration, we conducted genome-wide transcriptomic analysis of wound tissues using RNA sequencing (RNA-seq) (Figure 8A). Heatmap visualization of differentially expressed genes demonstrated that 3D-NEBS treatment markedly upregulated genes involved in angiogenesis (e.g. *Apln*, *VEGFA*), collagen biosynthesis (*Col1a1*, *Col1a2*), antioxidant defense (*Sod1*, *Hmox1*, *GPX3*) and anti-inflammatory immunomodulation (*IL4*, *Arg1*, *IL10*). Conversely, it downregulated pro-inflammatory mediators (*IL6*, *CD86*), apoptosis-related genes (*Bid*, *Casp3*) and pro-fibrotic markers (*Timp1*, *Tgfb1*). This expression profile indicates that 3D-NEBS facilitates healing through synergistic multi-target regulation of the diabetic wound microenvironment.

GO enrichment analysis further highlighted key biological processes orchestrated by 3D-NEBS (Figure 8B), including collagen fibril organization—critical for restoring mechanical strength and supporting tissue remodeling; skin morphogenesis—indicative of its role in promoting re-epithelialization and skin appendage regeneration; and modulation of immune response—consistent with facilitated M1-to-M2 macrophage polarization and inflammation resolution. KEGG pathway analysis revealed significant enrichment in type I diabetes mellitus and cell adhesion molecule pathways (Figure 8C), suggesting that 3D-NEBS may ameliorate insulin resistance and enhance cell–matrix communication to accelerate repair processes. In addition, the volcano plot (Figure 8D) visually presents the gene expression changes of the 3D-NEBS treatment group compared to the control group, revealing 689 significantly upregulated and 583 significantly downregulated transcripts, confirming the multitarget regulatory effect of 3D-NEBS on the microenvironment of diabetic wounds. Gene Set Enrichment Analysis (GSEA) corroborated these findings, showing pronounced activation of pathways related to collagen trimer assembly, extracellular matrix organization and positive regulation of interleukin-10 production (Figure 8E–G). These transcriptomic signatures align well with the observed phenotypic improvements in inflammation control, angiogenesis stimulation and structured collagen deposition, providing a comprehensive molecular basis for the therapeutic efficacy of 3D-NEBS in diabetic wound regeneration.

## Conclusion

In this study, we developed a multifunctional 3D-printed nanoparticle enhanced bionic skin (3D-NEBS) that synergistically combines antioxidative, anti-inflammatory, pro-angiogenic and antibacterial (including anti-biofilm) properties to address the complex microenvironment of diabetic wounds. By integrating the innate biological activities of *Tremella* polysaccharide (TP) with the robust catalytic functions of PDA-Ga-Arg nanoparticles within a dynamically crosslinked hydrogel network, 3D-NEBS promotes diabetic wound repair through several coordinated mechanisms: (i) TP contributes to moisture retention and intrinsic ROS scavenging; (ii) nanoparticles enable pH-responsive modulation of the ROS–NO balance, enhancing angiogenesis and mitigating oxidative damage; and (iii) the Schiff base-crosslinked hydrogel offers mechanical adaptability, self-healing behavior and a biocompatible matrix conducive to cell adhesion and tissue integration.

Studies collectively demonstrated that 3D-NEBS significantly accelerates wound closure, promotes collagen deposition, stimulates vascularization and reprograms macrophage polarization from M1 to M2 phenotype. Transcriptomic profiling further revealed upregulation of pathways related to angiogenesis, collagen assembly, antioxidant defense and anti-inflammatory signaling, providing deep molecular-level insight into its regenerative functions.

Despite these promising results, certain limitations should be acknowledged. The translational potential of this system would benefit from further investigation into the long-term biosafety and metabolic fate of nanoparticles components *in vivo*. Moreover, while the hydrogel demonstrated excellent printability and mechanical performance, scaling up the fabrication process to meet clinical-grade manufacturing standards requires additional optimization. Finally, although transcriptomic data strongly support multitarget mechanisms, further functional validation using gene-editing or inhibitor-based approaches would strengthen the causal inference of key signaling pathways. Importantly, the controlled release of Ga³^+^ from the PDA-Ga-LArg nanoparticles remained within a safe margin relative to known toxicity thresholds, as supported by both release kinetics and systemic biosafety assessments. This underscores the clinical translational feasibility of 3D-NEBS as an advanced and safe strategy that may also be adaptable to other conditions characterized by oxidative stress, impaired angiogenesis and persistent inflammation.

Notwithstanding these limitations, this study highlights the considerable potential of integrating natural polymers with functional nanoparticles for the treatment of chronic diabetic wounds. 3D-NEBS represents an advanced and clinically promising strategy that may also be adaptable to other conditions characterized by oxidative stress, impaired angiogenesis and persistent inflammation.

## Funding

This work was grateful for the support from the Special Foundation for Basic Research Program of Jiangsu Province (Soft Science Research; Grant BK20240490) and the Project of Affiliated Hospital of Nantong University (Tdb2122).

## Supplementary Material

rbag066_Supplementary_Data

## Date availability

The datasets generated and/or analyzed during this study are available from the corresponding author on reasonable request.
