# Supplementary material for: Three-dimensional-printed ROS-scavenging and immunomodulatory hydrogel accelerates diabetic wound healing through synergistic microenvironment regulation
Source: Regen Biomater. 2026 Mar 28;13:rbag066. doi: 10.1093/rb/rbag066 (PMC13110001; doi:10.1093/rb/rbag066)
Supplement: rbag066_Supplementary_Data [file rbag066_supplementary_data.docx]

**Supporting information**

**3D-Printed ROS-Scavenging and Immunomodulatory Hydrogel Accelerates Diabetic Wound Healing through Synergistic Microenvironment Regulation**

*Xin Cao, ^a, b, 1^ Yang Wang, ^c 1^ Yuanhang Xu, ^d 1^Nai Liang, ^e^ Jiabao Xu, ^f^ Xinyun Li, ^f^ Cong Ye ^a, *^ and Caichou Zhao ^a, *^*

^a^ Department of Dermatology, Affiliated Hospital of Nantong University, Medical School of Nantong University, Nantong 226001, China

^b^ Affiliated Hospital of Hebei University, Baoding 071000, China.

^c^ The Third People's Hospital of Qidong, Qidong 226200, China.

^d^ Dongyang People's Hospital, Dongyang 322100, China.

^e^ Maternal and Child Health Hospital of Changxing County, Changxing 313100, China.

^f^ Affiliated Hospital of Nantong University, Medical School of Nantong University, Nantong 226001, China

^1^ These authors contributed equally to this work.

^*^ Corresponding authors.

E-mail: cczhao@cmu.edu.cn (C.Z.);

gloryyezi@outlook.com (C. Y.);

**Supplementary Experimental Results**

**Table S1.** Blood Biochemistry and Routine Hematological Parameters on Day 14

|  | **Group** | | | | **Reference Range** |
| --- | --- | --- | --- | --- | --- |
|  | Control | Hydrosorb^®^ | 3D-BS | 3D-NEBS |  |
| AST (U/L) | 101 ± 6 | 94± 8 | 141 ± 12 | 119 ± 19 | 84 – 152 |
| ALT (U/L) | 28 ± 2 | 34 ± 3 | 35 ± 3 | 40 ± 3 | 25– 44 |
| TP (g/L) | 53 ± 2 | 59 ± 3 | 58 ± 2 | 63 ± 3 | 53 – 70 |
| ALB (g/L) | 26 ± 1 | 33± 1 | 30 ± 2 | 34 ± 2 | 24 – 36 |
| A/G | 1.2 ± 0.1 | 1.1 ± 0.1 | 1.3 ± 0.1 | 1.1 ± 0.1 | 0.6 – 1.2 |
| BUN (mmol/L) | 6± 2 | 7 ± 2 | 6 ± 1 | 8 ± 2 | 3–11 |
| Crea (umol/L) | 19 ± 2 | 33 ±3 | 35 ± 2 | 39± 5 | 16 – 44 |
| RBC (*10^12^ /L) | 4 ± 1 | 6 ± 1 | 7 ± 1 | 5 ± 1 | 4 – 10 |
| WBC (*10^9^ /L) | 7 ± 3 | 7 ± 1 | 6 ± 1 | 5 ± 2 | 4 – 12 |
| PLT (*10^9^ /L) | 1106 ± 124 | 1015 ± 88 | 1091 ± 21 | 1029 ± 105 | 737 –1342 |

AST: Aspartate aminotransferase, ALT: Alanine aminotransferase, TP: Total protein, ALB: Albumin, A/B: Albumin globulin ratio, BUN: Blood urea nitrogen, Crea: Creatinine, RBC: Red blood cells, WBC: White blood cells, PLT: Platelets


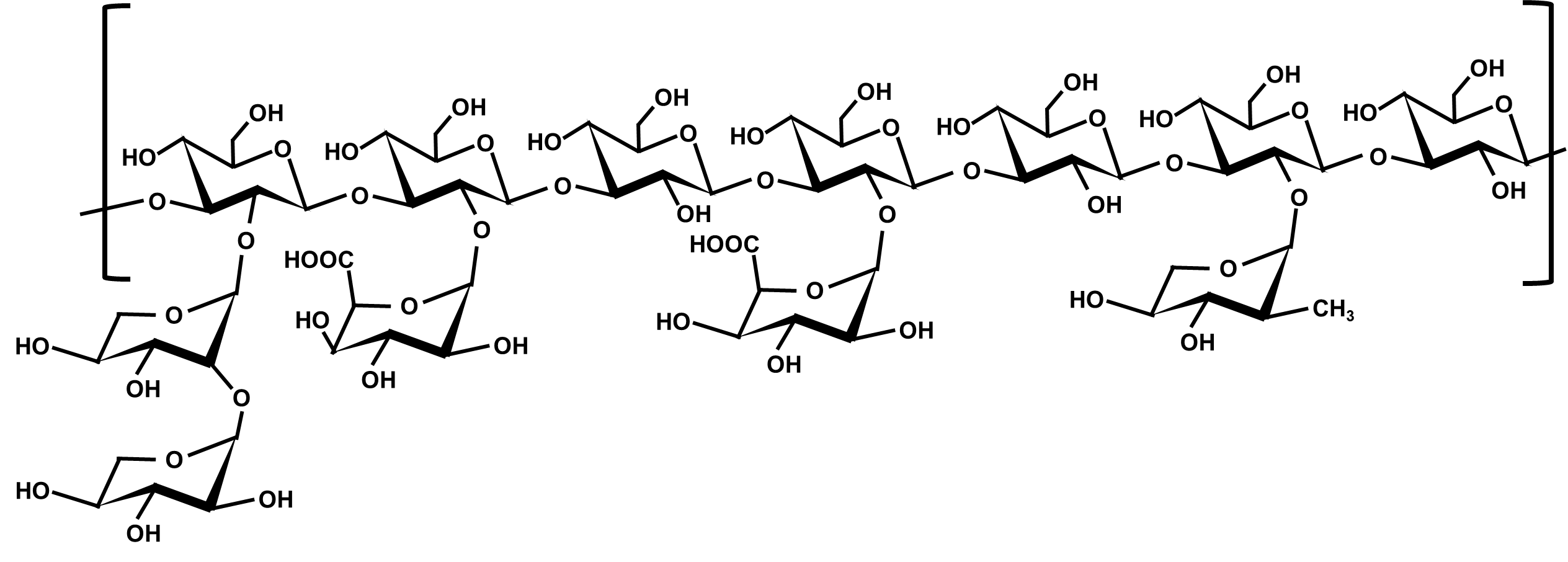


**Figure S1.** Molecular formula of TP.


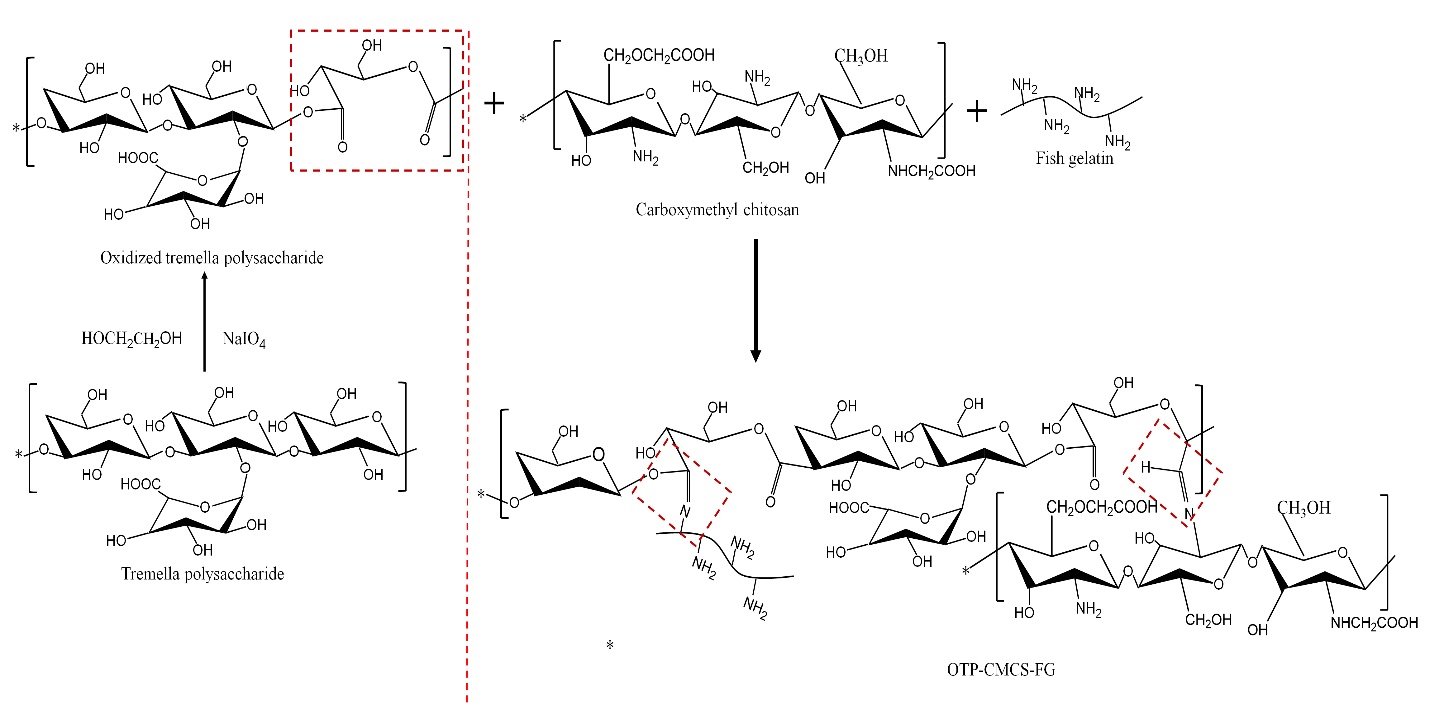


**Figure S2.** Schematic illustration of the synthesis process of OTP-CMCS-FG self-healing hydrogel.


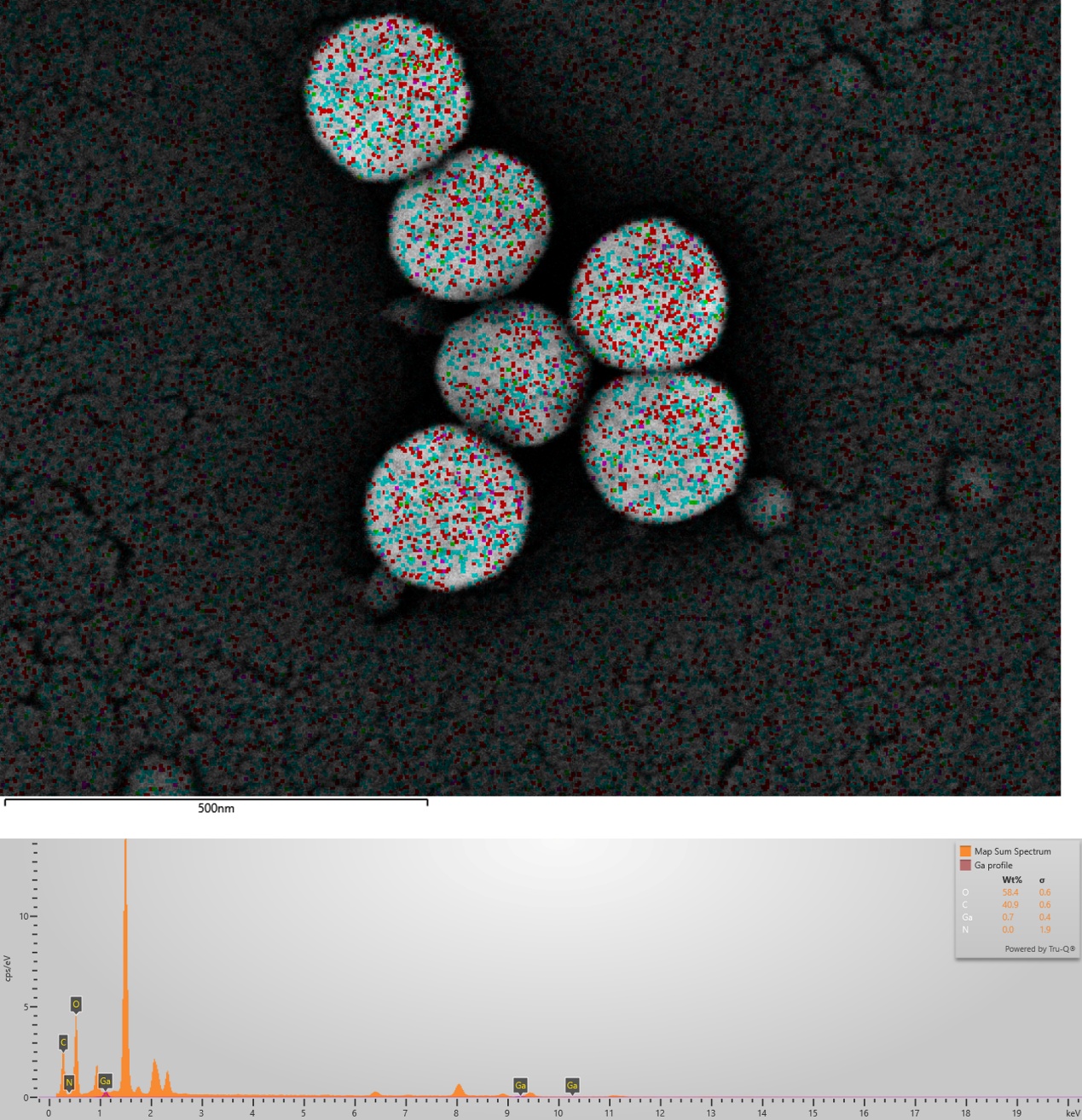


**Figure S3.** EDS analysis of PDA-GA-LARG.


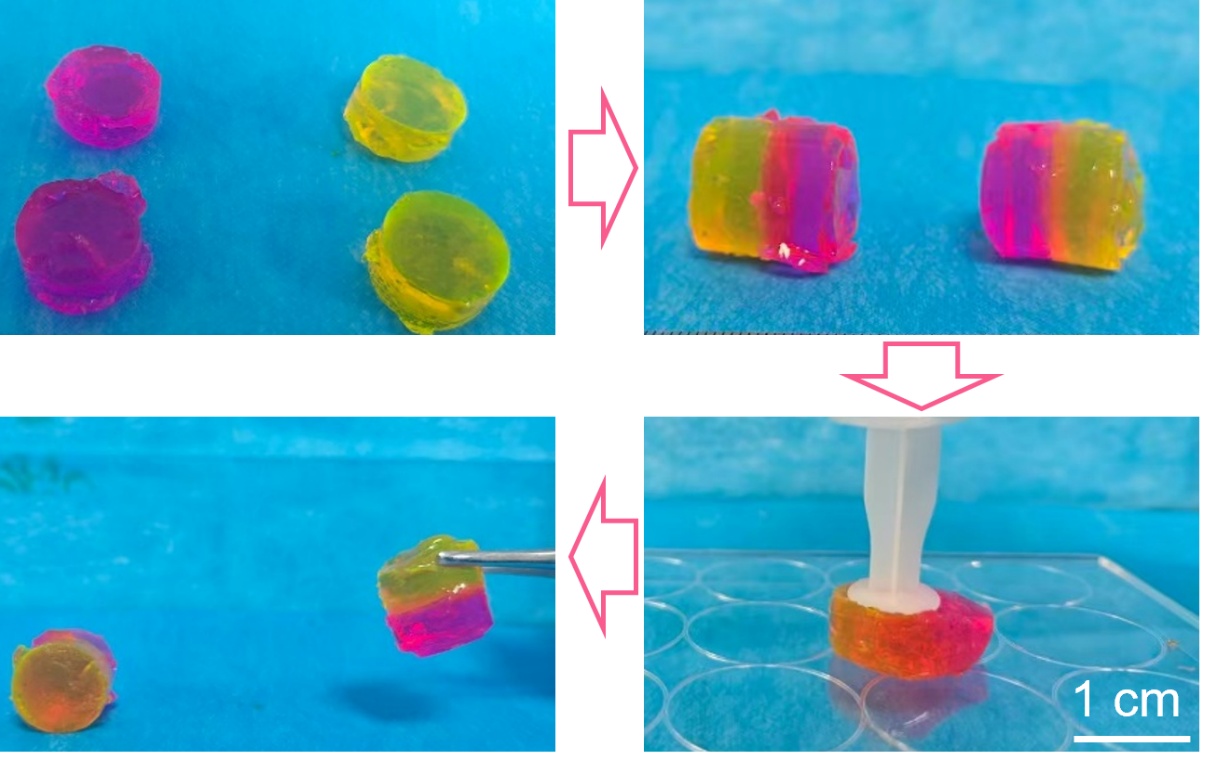


**Figure S4.** Demonstration of the self-healing property of the OTP/CMCS/FG composite hydrogel.


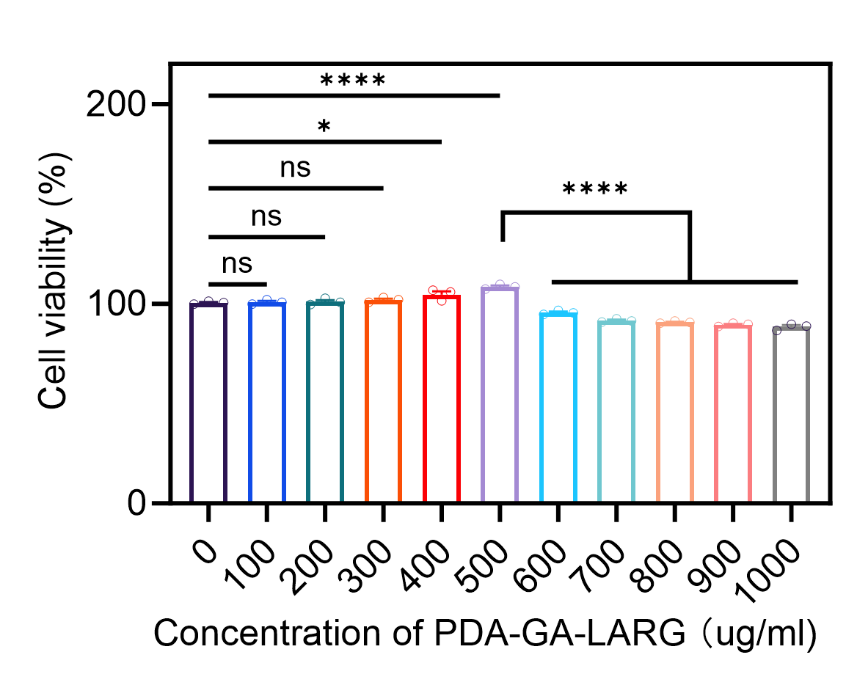


**Figure S5.** Viability of HUVECs after 24 h treated with PDA-GA-LARG nanozymes (n = 3). The data are presented as means ± SDs; statistical differences were assessed using one-way analysis of variance (ANOVA) with Tukey’s post hoc test; ns: not significant. * p < 0.05, ** p < 0.01, *** p < 0.001 and ****p < 0.0001.


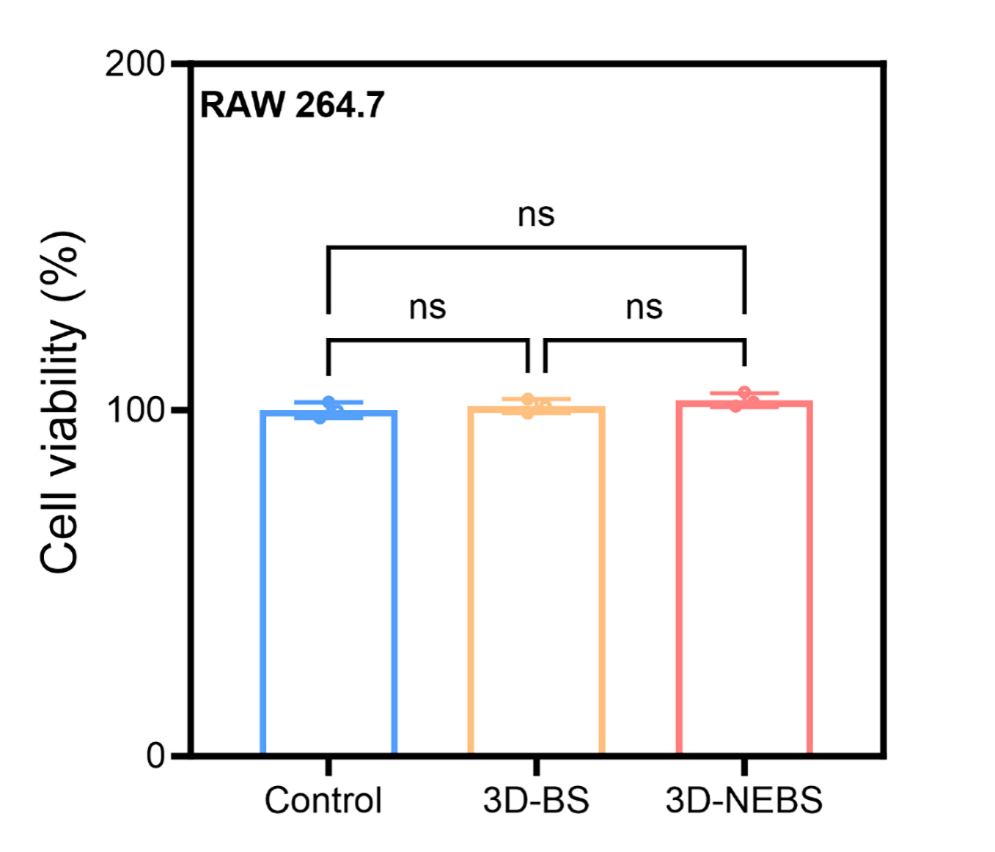


**Figure S6.** The CCK-8 analysis of macrophages treated with different hydrogels for 24 h. The data are presented as means ± SDs; statistical differences were assessed using one-way ANOVA with Tukey’s post hoc test; ns: not significant.


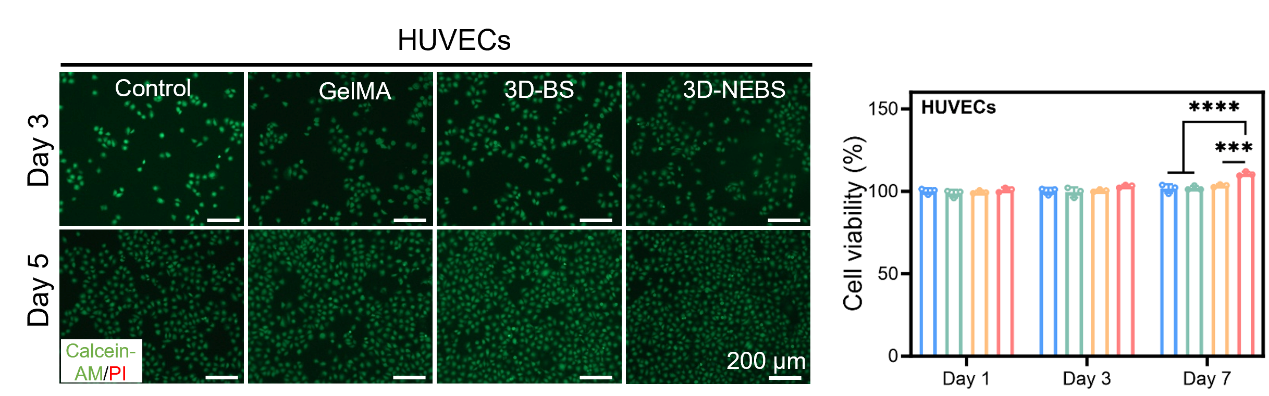


**Figure S7.** Live/dead staining analysis was performed on human umbilical vein endothelial cells that had been treated with different hydrogels for 3 days and 5 days, and CCK-8 analysis was conducted on these cells at 1 day, 3 days, and 7 days. The data are presented as means ± SDs; statistical differences were assessed using one-way ANOVA with Tukey’s post hoc test; ns: not significant. *** p < 0.001 and ****p < 0.0001.


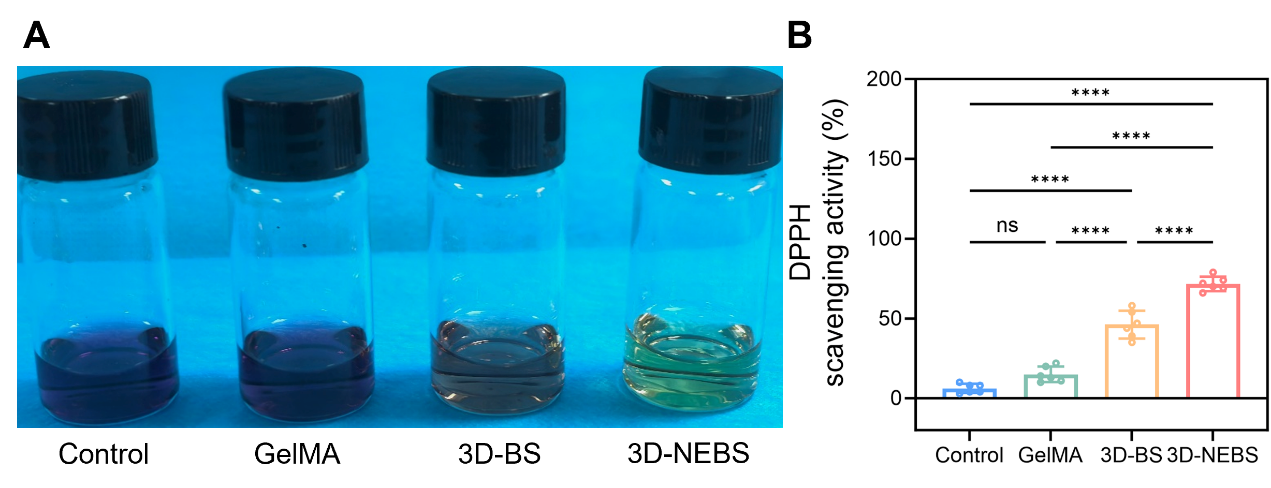


**Figure S8.** (A) Macroscopic images of 2,2-diphenyl-1-picrylhydrazyl (DPPH) radical scavenging assay. (B) Quantitative analysis of antioxidant activity (n=6), demonstrating dose-dependent free radical elimination. The data are presented as means ± SDs; statistical differences were assessed using one-way ANOVA with Tukey’s post hoc test; ns: not significant, *p < 0.05, **p < 0.01, ***p < 0.001, ****p < 0.0001.


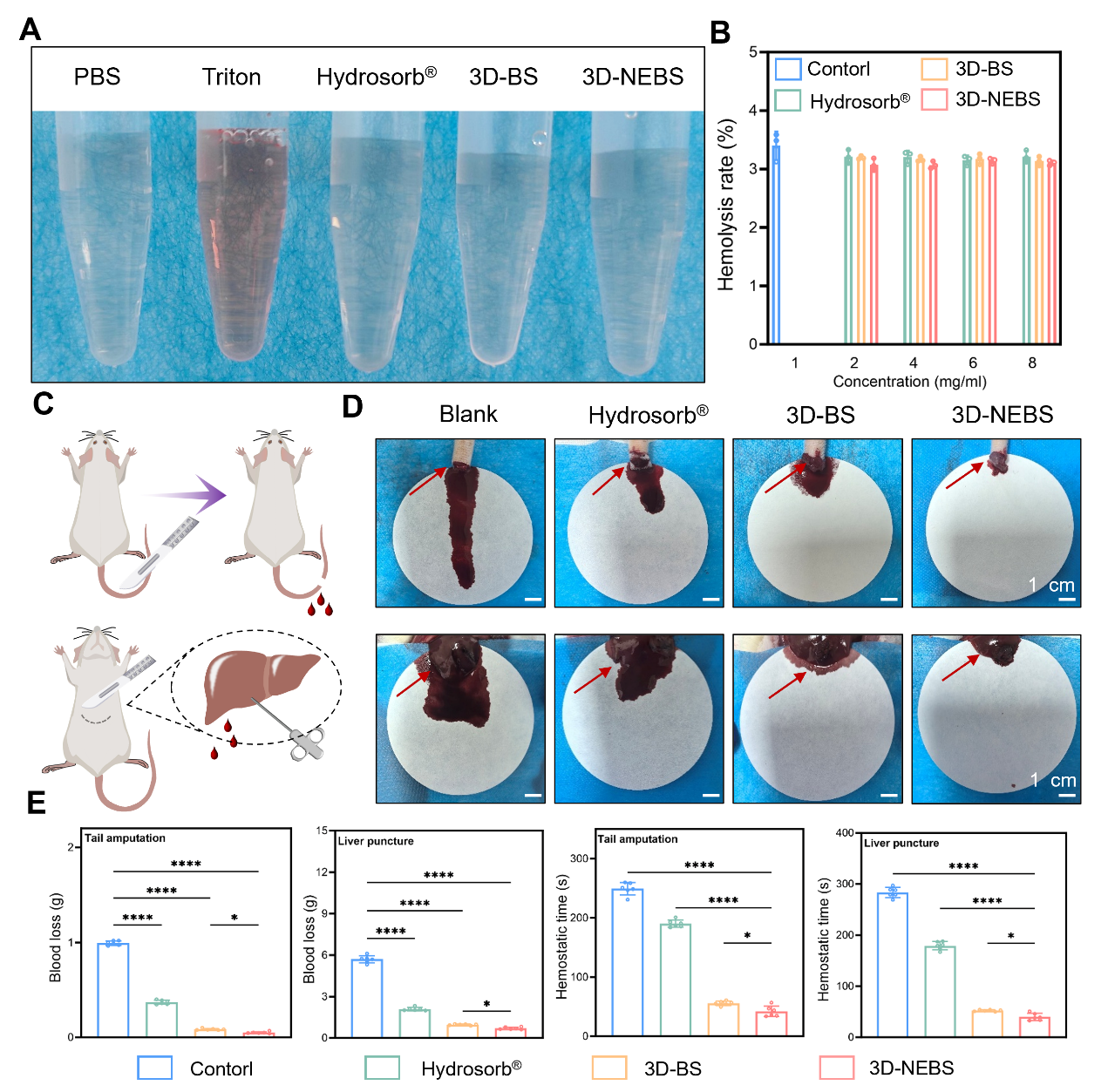


**Figure S9.** (A) digital images of hemolysis; (B) statistical data of hemolysis experiments; (C-E) Tail transection and liver puncture models were established in SD rats, and the hemostatic performance was evaluated using absorbable medical gelatin (Hydrosorb^®^), 3D-BS and 3D-NEBS. Scale bar: 1 cm. Data are presented as mean ± standard deviation (n = 6). statistical differences were assessed using one-way ANOVA with Tukey’s post hoc test; * p < 0.05, ** p < 0.01, ***< p < 0.001 and ****p < 0.0001.


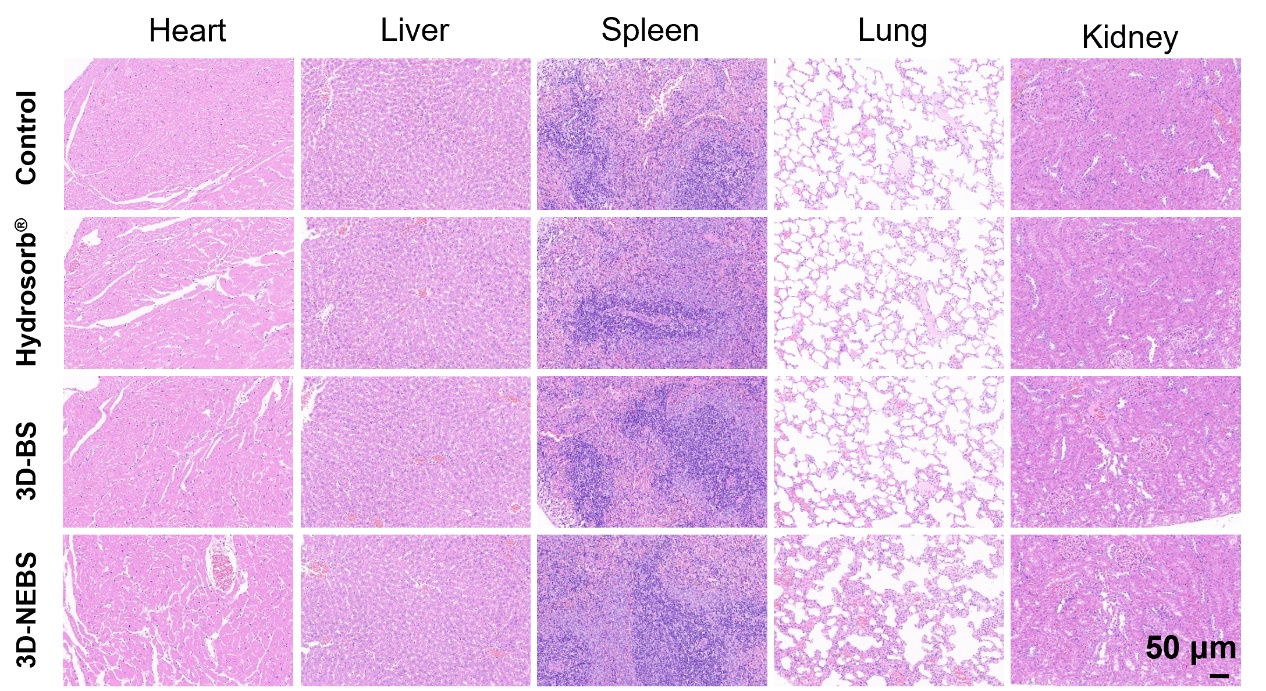


**Figure S10.** H&E Staining Images of Major Organs from Rats with Diabetic back wounds Receiving Different Treatments on Day 14.


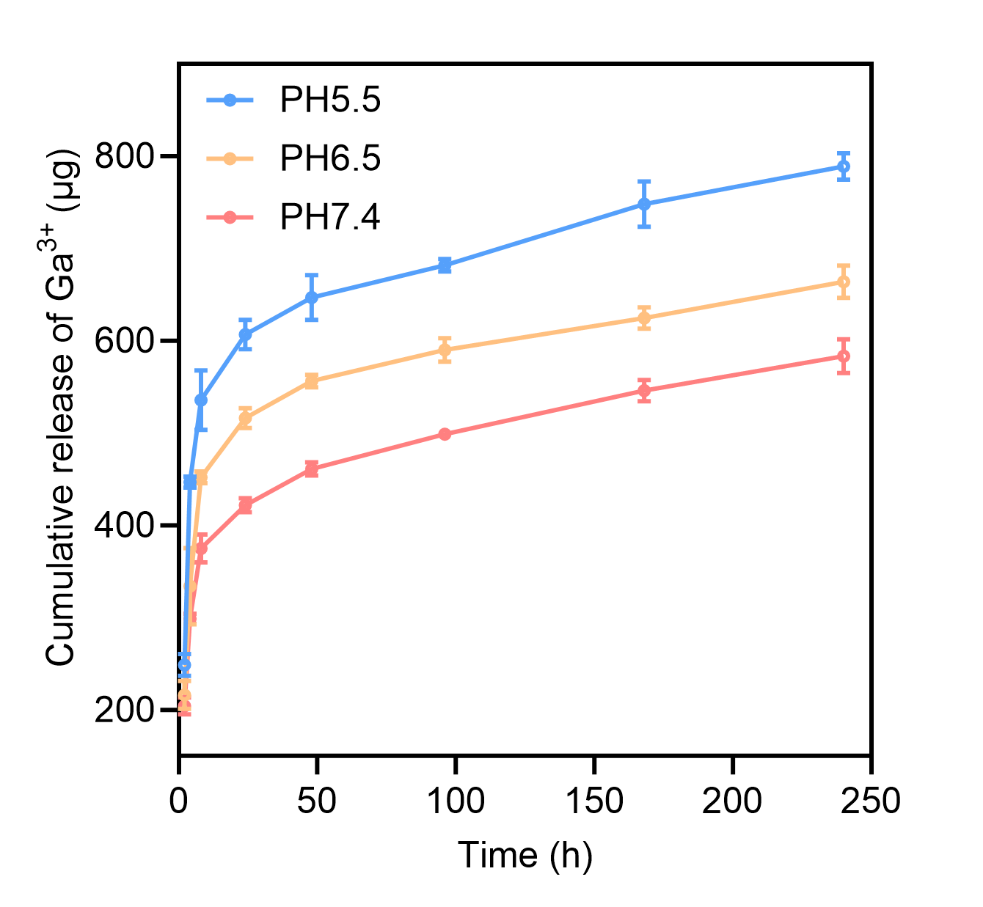


**Figure S11.** The relationship between the cumulative Ga release amount and time in 3D-NEBS.


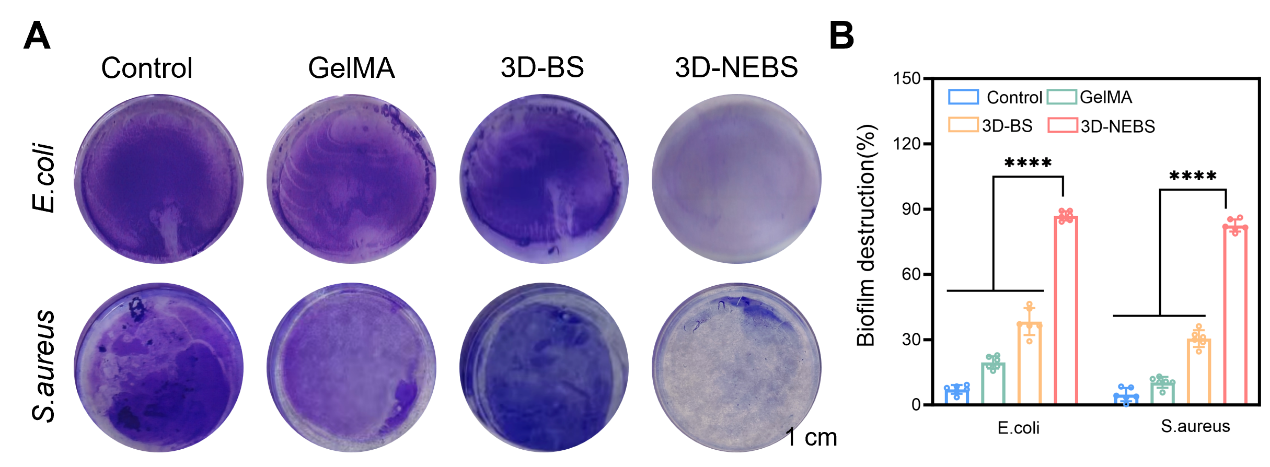


**Figure S12.** (A) Macroscopic image of crystal violet staining. (B) Statistical graph of the antibiofilm test results. The data are presented as means ± SDs; statistical differences were assessed using one-way ANOVA with Tukey’s post hoc test; ns: not significant, ****p < 0.0001.


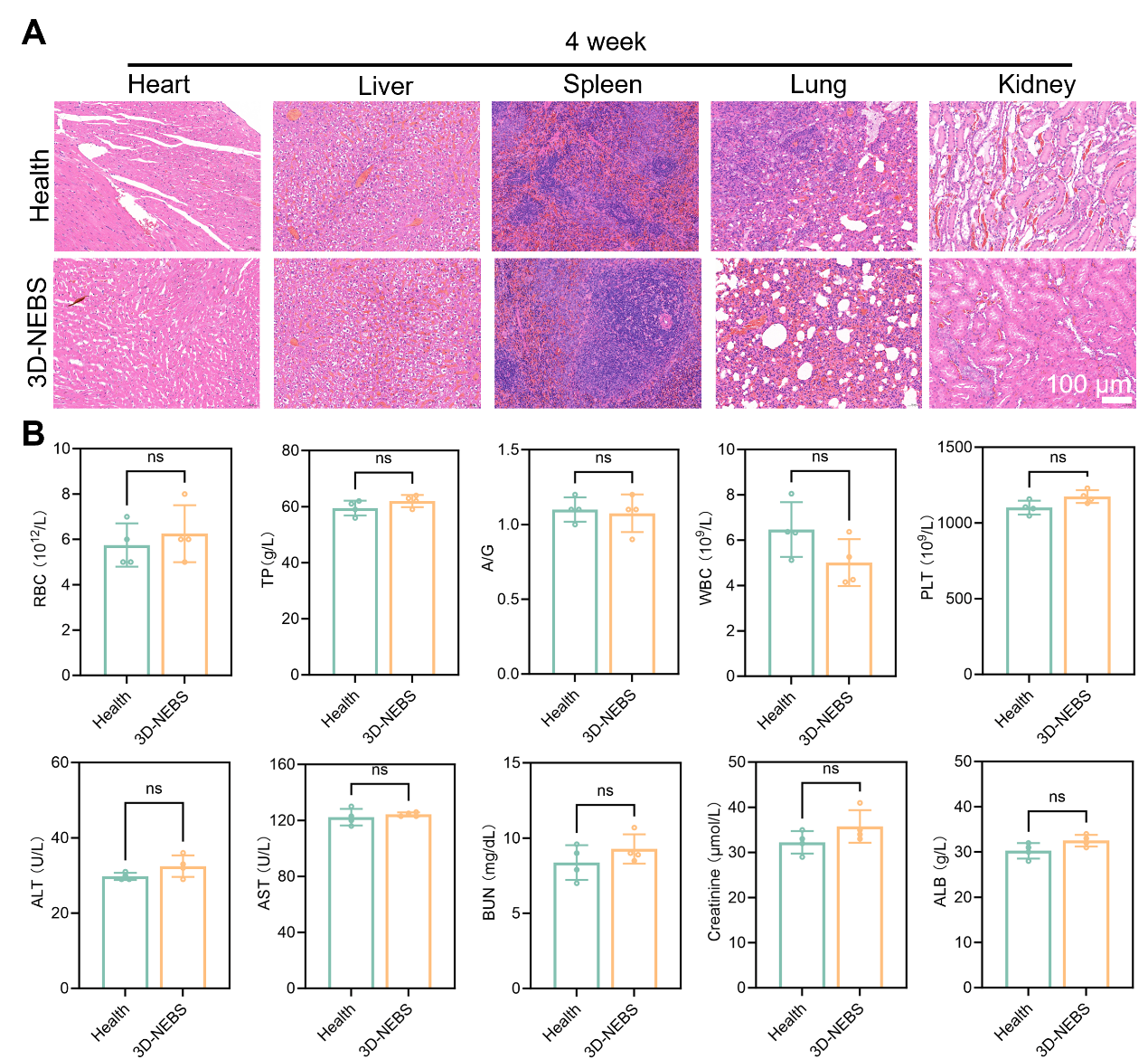


**Figure S13.** HE staining of the main organs and analysis of the main blood biochemical indicators in the body 4 weeks after 3D-NEBS was implanted into the abdominal cavity; (A) statistical 3D-NEBS and HE staining of major organs in normal rats. (B) Results of blood biochemical indicators in the fourth week (n=4). AST: Aspartate aminotransferase, ALT: Alanine aminotransferase, TP: Total protein, ALB: Albumin, A/B: Albumin globulin ratio, BUN: Blood urea nitrogen, Crea: Creatinine, RBC: Red blood cells, WBC: White blood cells, PLT: Platelets. The data are presented as means ± SDs; Use t-tests (and non-parametric tests) for pairwise comparisons to evaluate statistical differences; "ns" indicates "no significant difference".

.


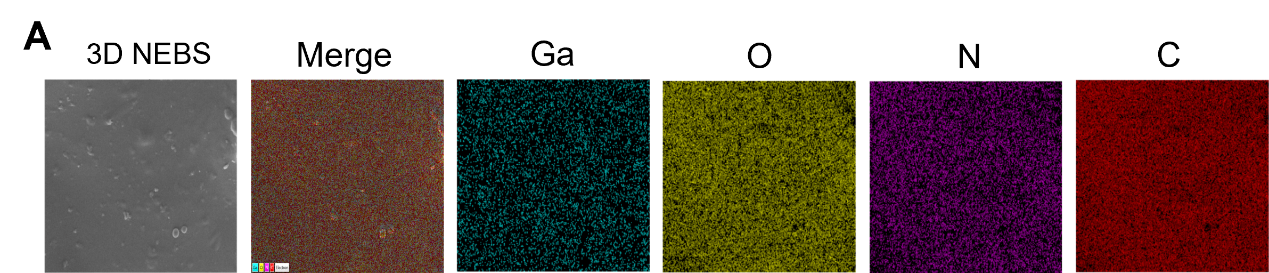


**Figure S14.** Elemental mapping analysis of 3D-NEBS confirms the uniform distribution of components.


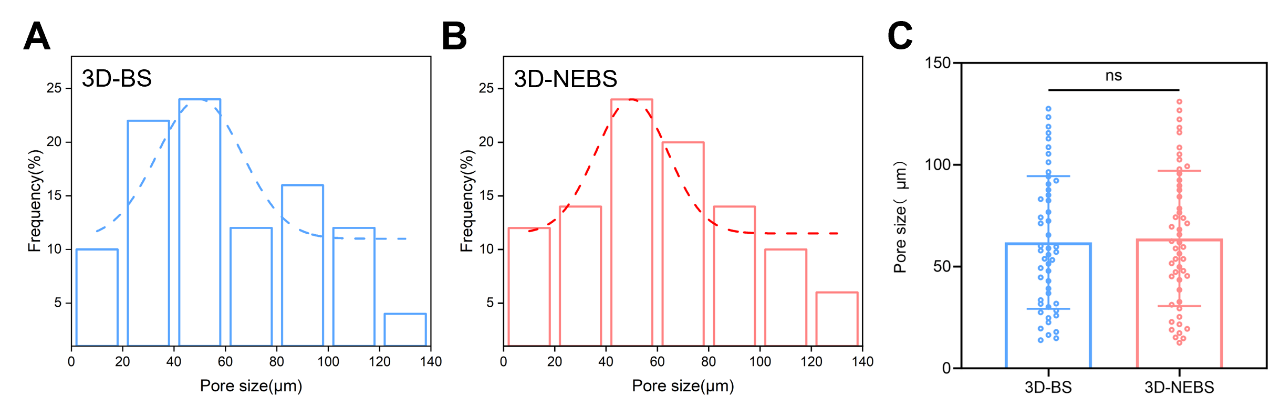


**Figure S15.** (A-B) Mean pore size, pore size distribution (e.g., D50, standard deviation), and porosity percentage. (C) Statistical comparison of pore structure between 3D-BS (without nanoparticles) and 3D-NEBS (with nanoparticles). The data are presented as means ± SDs; Use t-tests (and non-parametric tests) for pairwise comparisons to evaluate statistical differences; "ns" indicates "no significant difference".


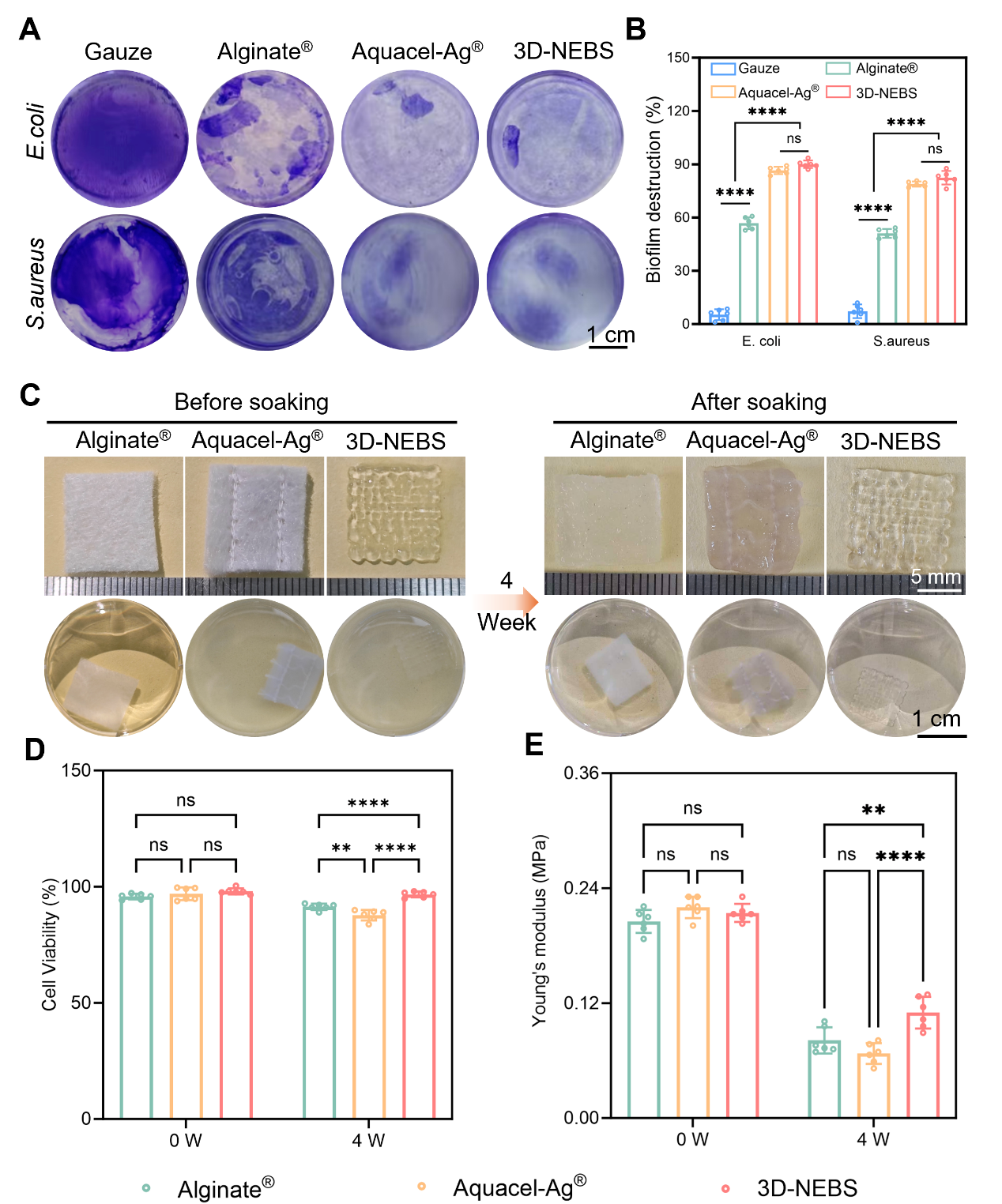


**Figure S16.** Long-term Stability, Mechanical Durability, and Functional Superiority of 3D-NEBS Compared to Clinical-Grade Wound Dressings. (A). Macroscopic image of crystal violet staining (clinical-grade wound dressings (Gauze, Alginate^®^, Aquacel-Ag^®^) and 3D-NEBS. (B). Quantitative analysis of antibacterial efficacy (E. coli and S. aureus) of different dressings, presented as mean ± SD (n=6) (C). Gross morphological observation of Alginate^®^, Aquacel-Ag^®^, and 3D-NEBS, demonstrating shape retention before and after 4 weeks of SBF immersion. (D). Cytocompatibility evaluation of HUVECs co-cultured with SBF (collected after 4 weeks of dressing immersion), represented as relative cell viability (n=6). (E). Young's modulus of various dressings after 4 weeks of SBF immersion, measured by tensile testing (n=6). The data are presented as means ± SDs; statistical differences were assessed using one-way ANOVA with Tukey’s post hoc test; ns: not significant, * p < 0.05, ** p < 0.01, ***< p < 0.001 and ****p < 0.0001.


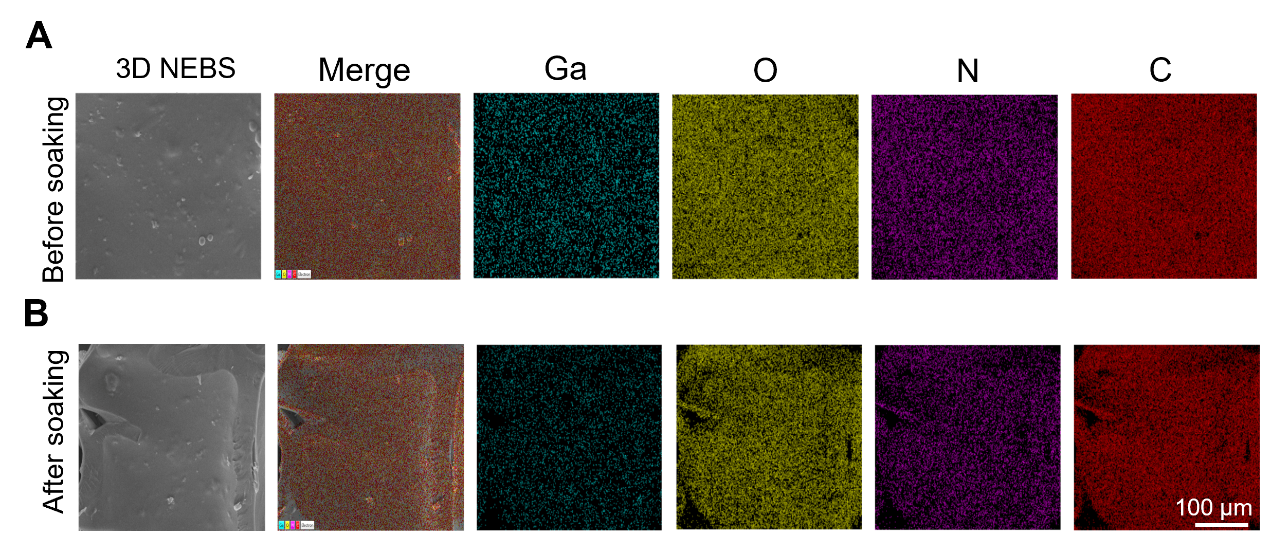


**Figure S17.** Comparison of the distribution of nanoparticles on 3D-NEBS after 4 weeks of immersion in simulated body fluid.

**Table S1. 3D printing parameters**

| **Parameters** | **Value** |
| --- | --- |
| Nozzle temperature (℃) | 16 |
| Line distance/wire diameter | 200μm/200μm |
| Print Speed (mm/s) | 5 |
| Extrusion Speed (mm^3^/s) | 0.72 |
| Height of Patches (mm) | 0.6 |
